# iMAP: A Web Server for Metabolomics Data Integrative Analysis

**DOI:** 10.3389/fchem.2021.659656

**Published:** 2021-05-05

**Authors:** Di Zhou, Wenjia Zhu, Tao Sun, Yang Wang, Yi Chi, Tianlu Chen, Jingchao Lin

**Affiliations:** ^1^Metabo-Profile Biotechnology (Shanghai) Co. Ltd., Shanghai, China; ^2^Center for Translational Medicine, Shanghai Jiao Tong University Affiliated Sixth People's Hospital, Shanghai, China

**Keywords:** metabolomics, statistical analysis, workflow, correlation-based network, visualization

## Abstract

Metabolomics data analysis depends on the utilization of bioinformatics tools. To meet the evolving needs of metabolomics research, several integrated platforms have been developed. Our group has developed a desktop platform IP4M (integrated Platform for Metabolomics Data Analysis) which allows users to perform a nearly complete metabolomics data analysis in one-stop. With the extensive usage of IP4M, more and more demands were raised from users worldwide for a web version and a more customized workflow. Thus, iMAP (integrated Metabolomics Analysis Platform) was developed with extended functions, improved performances, and redesigned structures. Compared with existing platforms, iMAP has more methods and usage modes. A new module was developed with an automatic pipeline for train-test set separation, feature selection, and predictive model construction and validation. A new module was incorporated with sufficient editable parameters for network construction, visualization, and analysis. Moreover, plenty of plotting tools have been upgraded for highly customized publication-ready figures. Overall, iMAP is a good alternative tool with complementary functions to existing metabolomics data analysis platforms. iMAP is freely available for academic usage at https://imap.metaboprofile.cloud/ (License MPL 2.0).

## Introduction

Metabolomics data analysis depends on specialized bioinformatics tools. After raw data pre-processing (peak finding, matching, and quantifying), metabolomic data analysis can be summarized as three steps. Step 1: data-processing including data filtering and normalization. Step 2: metabolites-selection. In this step, statistical analysis will be performed to identify/select metabolites that are significantly changed between/among groups or are significantly correlated with phenotypes. Step 3: data-interpretation. The aim of this step is to reach a biologically significant conclusion from previous results (Wanichthanarak et al., 2017). Plenty of open-source platforms, including Metaboanalyst (Chong et al., 2018), W4M (Giacomoni et al., 2015), Galaxy-M (Davidson et al., 2016), XCMS-online (Huan et al., 2017), MZmine2 (Pluskal et al., 2010), MetaBox (Wanichthanarak et al., 2017), and MS-DIAL (Tsugawa et al., 2015) have been developed and successfully used for metabolomics data analysis. There are usually two usage modes, predefined workflows and independent modules, taken in these platforms with their advantages and disadvantages. Comparatively, the predefined workflows are simpler with complete (or nearly complete) functions and recommended parameters. It is more preferred for batch analysis or preliminary analysis. The independent modules are more flexible. Users can select one or some of the modules they want and get quick or refined results.

We have developed IP4M (Liang et al., 2020) for metabolomics data mining which covers all the key steps from pre-processing to data-interpretation and can be freely accessed via https://github.com/IP4M. Given the new trends of metabolomics data analysis and to meet the evolving needs of IP4M users, we improved the modules and workflows and developed a webserver named integrated Metabolomics Analysis Platform (iMAP). Compared with IP4M, iMAP provides more optional methods and workflows with more adjustable parameters and improved figures. New modules for predictive model building and validation and correlation-based network construction and analysis have been added. Modules of raw data preprocessing and peak picking and annotation were not implemented considering the risk of data security and the burden of big data uploading. In terms of usage mode, besides the aforementioned “independent modules” and “predefined workflows” iMAP also supports “customized workflows” mode. Modules belong to steps 1–3 can be incorporated into a thread-like or tree-like workflow freely by users according to their study aims and data characteristics. The user-defined workflows and relevant parameters can be saved for later analysis. This mode is particularly suitable for batch analysis or studies with special requirements. In summary, iMAP is a more mature, comprehensive, and modern platform to empower the metabolomics community. Here, we present the functions and applications of iMAP with the focuses on newly-added modules and workflows.

## Materials and Methods

**Figure 1:**
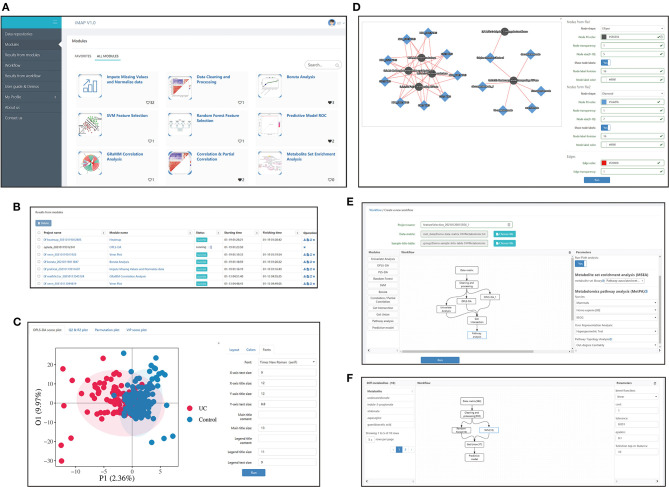
Interfaces screenshots of iMAP. **(A)** The main menu and part of modules. The main menu lies on the left. And modules are shown on the right after clicking “Modules” in the main menu. Over 20 modules are available in iMAP at present. Click “User guide & Demos” to check detailed manuals. **(B)** The interface showing results from modules. Users can view detailed results, change parameters for visualization, download the results or rerun the module with modified parameters. Interface showing the results from workflows working in the same way. **(C)** Demonstration of the visual parameter in OPLS-DA result. Users can change parameters for layout (such as whether to show labels and confidence ellipse, picture size), choose colors, and adjust font parameters. **(D)** Demonstration of the visual parameter in a correlation-based network. All plot results from modules and workflows can be modified like the OPLS-DA results and the correlation-based network, designed to provided users with high-quality, publishable plots. **(E)** The workflow creation interface. After the data matrix and sample information table were chosen, modules in the left panel can be dragged into the center panel. Users can construct their customized workflow by connecting modules with arrows. Parameters can be modified in the right panel when a module was selected. **(F)** The interface of detailed workflow results. Users can see the workflow in the center panel. The number in brackets showed the count of the selected metabolites by each module. The selected module's outline will turn blue like the “SVM” module in the center panel. Users can click on a module to check the parameters used in the right panel. Users can also click on the module and check the metabolites selected by that module on the left panel. The 10 metabolites selected by the SVM module, including undecanedionate, indoe-3-propionate, etc., were shown in the left panel.

iMAP is an R-based free web application. Users can mine their data by either running the modules separately or running a customized workflow composed of multiple modules. Screenshots of some iMAP interfaces are shown in Figure 1. The main menu, available modules, interface for plot modification, interface for workflow construction, and interface for workflow result review are shown in Figure 1. The online “User guide & Demos” in iMAP provides more detailed information.

All the resulting data files of iMAP are in CSV format and hence can be imported directly (or after minor revision) to many existing platforms for further analysis. Users are advised to check and download their results in a timely manner. We will preserve up to 100 module results and 100 workflow results for each user, for up to 1 month.

### Input Data

The following three types of files can serve as the “Input data”:

(1) A data matrix of preprocessed peaks intensity or compounds concentration.
(2) Sample information table with SampleID and group and other alternative information (such as pair_ID in paired groups study, time in repeated measurement, clinical information for patients, phenotype etc.).
(3) Variable information table with variable names and variable features such as class, or subclass of metabolites. The variable information table is an optional input file.

Demonstration input data for workflow was provided and was available in the popped-up window (Supplementary Figure 2A) after clicking the “Choose file” button in Figure 1E. Demonstration input data for every single module in Figure 1A was also provided and was available in a similar way (Supplementary Figure 2B). Demonstration input data was also available in the “Data repositories” in Figure 1A. Users can access the input data and check the format by clicking “Upload file” -> “Using the sample file” (Supplementary Figure 2C).

### Data-Processing

In the workflow, the input data will first go through a data-processing module including highlighting missing value, removing samples/variables which have more missing values than the thresholds, filling up the missing value, normalization, batch effect correcting, remove unstable variables according to quality control samples, selecting variables by their absolute value, transformation, and scaling. Three data-processing examples summarized from published metabolomic studies were provided for users to choose (Supplementary Figure 1). Users can also change order and activated/deactivated steps or modify the parameters to build their customized data-processing strategies. A basic statistics summary and a principal components analysis (PCA) will be performed to explore the data onto the end of the data-processing.

### Metabolite Selection

**Figure 2:**
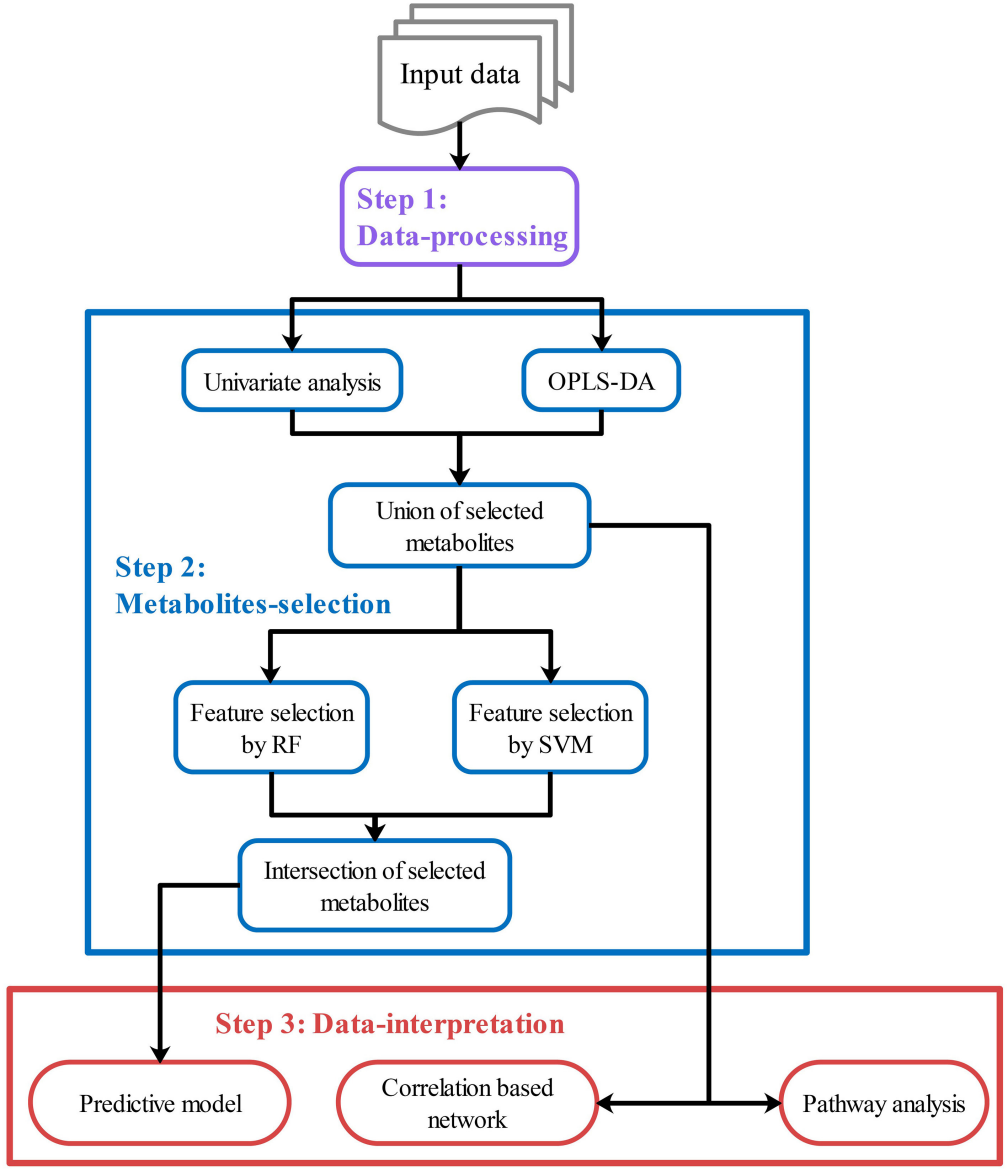
Customized analysis workflows demonstration. Customized workflow is constructed with modules in step 1–3, including data-processing modules (Step 1, purple), metabolites-selection modules (Step 2, blue), and data-interpretation modules (Step 3, red). Users can add/remove/edit/reorder modules in each step. Users can also skip any step if they do not need it.

After data-processing, statistical analysis will be performed in various ways to select key metabolites (“metabolites-selection”) including metabolites with a significant change between groups, metabolites with significant correlation with other data, or top-ranked metabolites in feature selection modules. Most of the commonly used methods in metabolomics studies were involved in the metabolites-selection section (Table 1). Analysis methods were packed in different modules. Figure 2 showed a typical workflow for metabolomics studies. The selected metabolites from univariate analysis and OPLS-DA were integrated. These selected metabolites can be directly passed to the pathway analysis module for result interpretation or be transferred to some feature selection modules (such as Random Forest or support vector machine). Users can add/remove/edit modules by clicking the mouse and connecting modules with arrows to control modules' execution order. After connecting two modules with an arrow, the metabolites used in the downstream module will be decided by the upstream module.

**Table 1:** Metabolites-selection methods provided by iMAP modules.

**Methods**	**Description**
Univariate analysis	Parametric and non-parametric test for two or more paired/independent groups.
Mutivariate analysis with dimensional reduction	PCA, PLS-DA and OPLS-DA
Other metabolite selection methods	RF, SVM, Boruta
Correlation-based network analysis	Correlation analysis Partial correlation analysis GRaMM Node centrality analysis
Pathway analysis	Metabolite set enrichment analysis (MSEA) Metabolomics pathway analysis (MetPA)
Model building and validation	RF, SVM, GB, Boruta, Logistic regression, Elastic Net

To integrate selected metabolites from different modules, specialized summary modules (the “Union of selected metabolites” module and the “Intersection of selected metabolites” module in Figure 2) were provided to get union sets or intersection sets. By connecting multiple upstream modules (such as the univariate analysis module and OPLS-DA in Figure 2) to a downstream summary module, users can integrate selected metabolites from different modules. The intersection or union sets metabolites can also change metabolites to be used in the downstream modules (such as “Feature selection by RF” in Figure 2). A variety of optional methods and parameters were provided for metabolites-selection.

### Univariate Analysis

Thresholds for P and FDR and |log2FC| can be set to select metabolites for analysis between two groups. When it comes to analysis among three (or more, here use three as an example) groups, P and FDR among three groups, thresholds for pairwise P and *post-hoc* P and |log2FC| can be set to select metabolites. Besides those commonly used thresholds, iMAP provided extra parameters named “trends-across-groups” to select metabolites. By setting the trend like “control -> low -> medium -> high,” metabolites which were sequential ascending or descending in control, low, medium, and high groups will be selected. In some study design, biomarkers meet the trends-across-groups might be more biologically interpretable (Sreekumar et al., 2009; Ramautar et al., 2013; Randall et al., 2013; Kim et al., 2017).

### Multivariate Analysis

OPLS-DA and PLS-DA analysis are commonly used multivariate methods for metabolites-selection in metabolomics studies. iMAP provided VIP for metabolites-selection in PLS-DA and OPLS-DA modules. To meet the increasing demand for controlling confounders and covariates in the metabolomics study, iMAP provided multivariate logistic regression to explore the adjusted relationship between groups and metabolites. Additionally, the commonly used feature selection methods in machine learning were also provided. Random forest, support vector machine, and Boruta can be utilized to select metabolites.

### Correlation and Partial Correlation

Correlation analysis and partial correlation analysis can also be utilized to select metabolites. Users can set thresholds for correlation coefficients and *P*-value for metabolites-selection. Partial correlation coefficients can be calculated in the partial correlation analysis module by controlling confounders and covariates.

Parametric and non-parametric tests between paired/independent groups, PLS-DA, OPLS-DA, RF, SVM, Boruta, correlation analysis, and partial correlation analysis are available to select metabolites in iMAP at present.

### Data Interpretation

After metabolites-selection, modules in data-interpretation can be performed to interpret the result by utilizing the metabolites selected from the upstream modules (Figure 2). Users can also build and validate a predictive model, or perform a correlation-based network analysis or do pathway analysis, including metabolite set enrichment analysis (MSEA) and metabolic pathway analysis (MetPA), by using pathway analysis modules.

### Customized Workflow

A customized workflow is a thread-like or tree-like diagram constructed by users according to their study aims. It can be constructed in the interface (Figure 1E) by dragging the wanted modules into the center panel and then connecting them by clicking the arrows on the modules. Users can modify the parameters of each module freely and save the workflow for later usage. There are seven example workflows provided in iMAP. They were summarized from published metabolomic studies with different strategies for key metabolites selection, pathway analysis, and/or predictive model building (Sreekumar et al., 2009; Liu et al., 2019; Bushman et al., 2020). It's convenient to construct customized workflows by modifying these example workflows. More information about the example workflows was provided in Supplementary Figure 1.

### Pipelines for Predictive Model Building and Validation

**Figure 3:**
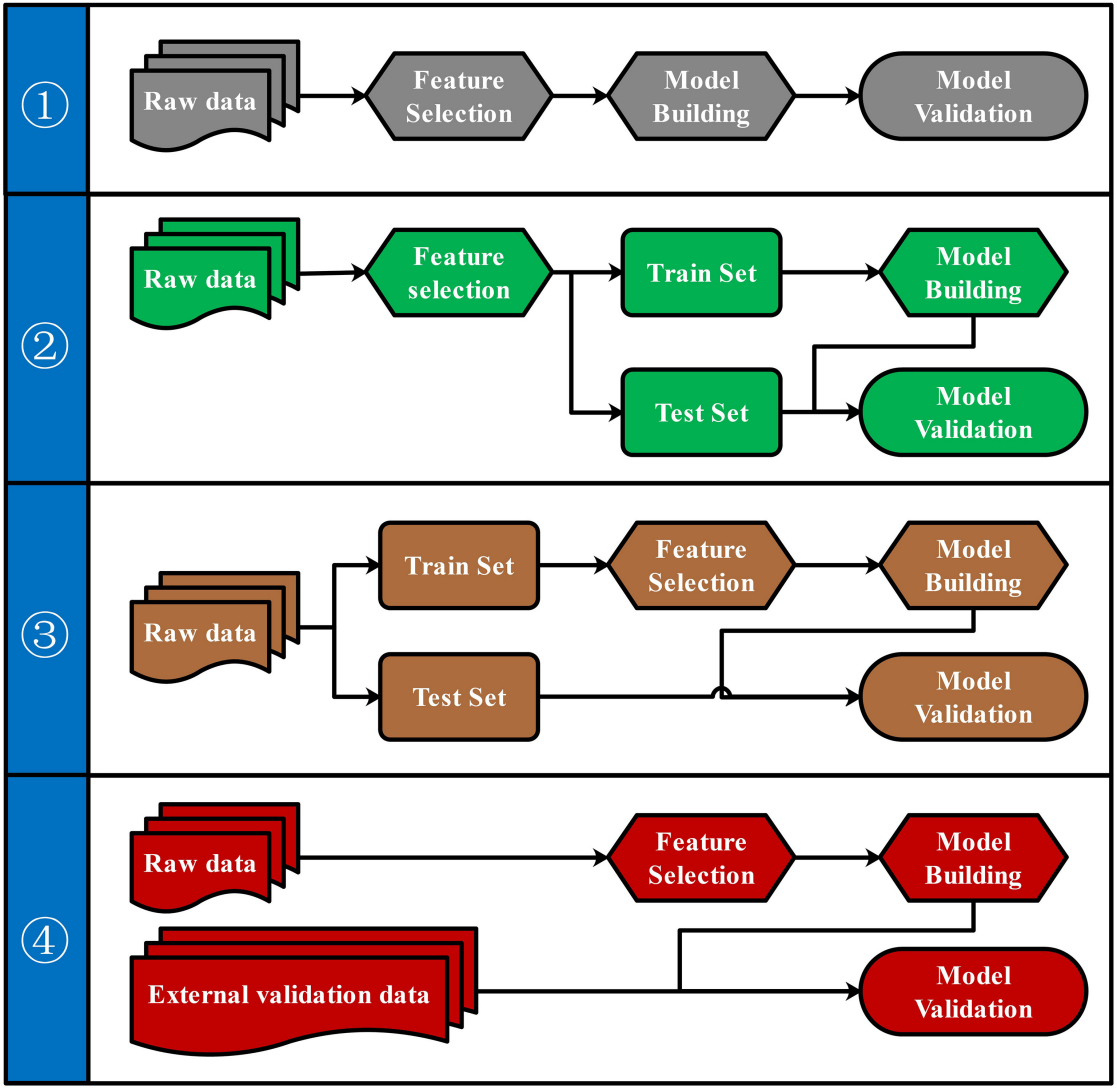
Four pipelines for the predictive model building. Four different pipelines for feature selection, model building, and model validation were shown.

To our knowledge, there were four commonly used pipelines for the predictive model building to diagnose diseases or to predict the prognosis (Figure 3). Pipeline build and validate the model based on the same data, thus might create an overly optimistic model and leave the risk of over-fitting (Ambroise and McLachlan, 2002; Cawley and Talbot, 2010). Pipeline seems to return a more objective evaluation of the model by split raw data and use different data set to build and validate the model separately. However, pipeline could lead to “data leakage” (also known as data snoop) because feature selection was performed before data separation (Kaufman et al., 2012). Because the raw data for feature selection in pipeline contains information from the test set, the selected features already have the test set's information. Thus, this information will leak to the predictive model. Data leakage makes the test set previewed by the model and may create an overly optimistic model. An overly optimistic model may exaggerate the model's predictive ability and may mislead and frustrate the following researchers. Pipeline could avoid data leakage by using the train set for feature selection. Furthermore, pipeline could verify the predictive model's robustness and generalization performance by importing external validation data.

Both biological and statistical experience were required to design a statistically appropriate analysis pipeline with a low risk of over-optimistic and/or over-fitting model. Programming skills may also be required to perform this pipeline with the high-dimensional metabolomics data. All four pipelines can be performed via iMAP workflow. All modules in “2.1.3 Metabolites selection” were available to select metabolites for predictive modeling. However, only pipeline and pipeline were recommended for more statistically appropriate conclusion. Users can activate a built-in parameter to separate raw data into train set and test set by stratified random sampling. Metabolites will be selected in step 2 by using train set data. After metabolites were selected, models will be built by the train set and validated by the test set to finish the predictive model via pipeline. Users can also perform pipeline by importing external validation data for model validation. Users newly to metabolomics can build their workflow by editing the default workflow provided. Experienced users can organize workflow according to their personalized demands. Thus, both green hand users and experienced users can conveniently design a customized and complicated workflow without coding.

### A Module for Correlation-Based Network Construction and Analysis

A correlation analysis involving omic data will generate vast results. It will be challenging and effort-consuming to summarize and interpret the vast correlation results. Correlation-based networks and graph-theory properties are commonly used in this condition and can help conquer this challenge (Batushansky et al., 2016). iMAP provided a correlation analysis module to meet these needs. The module can perform correlation (Pearson or Spearman) or partial correlation analysis and filter each pair of variables by thresholds (|*r*| > 0.5 and *P* < 0.05 by default) to get the edge table for the network. Other variables, including -ln(P), FDR, -ln(FDR), and the absolute value of correlation coefficient, will be calculated to map edges' visual features in the network. The node table will be generated from the edge table. The graph-theory centralities of nodes (including degree centrality, betweenness centrality, closeness centrality, and eigenvector centrality) will be calculated (Csardi and Nepusz, 2006). Centralities can reflect the importance of nodes and help users locate critical nodes in the network. Users can change parameters to control filtering algorithm and change mapping ways from variables to visual features. The module will create a network plot according to the user's setting. Users can also import the edge table and node table into Cytoscape to perform more personalized modifications.

### Implementations

The iMAP V1.0 was developed by Scala 2.13.1 (https://www.scala-lang.org/) and R 3.6.1 (https://www.r-project.org/). R packages including ropls, MetaboAnalystR, Hmisc, pROC, randomForest, and Boruta were utilized for analysis. The interactive build tool is SBT 1.3.5 (https://www.scala-sbt.org/). Web server is AKKA 2.6.5 (https://www.akka-technologies.com/) and database server is PostgreSQL 9.5 (https://www.postgresql.org/). The interfaces were designed and implemented using the Play Framework 2.8.2 (https://www.playframework.com/) and Bootstrap 3.3.0 (https://getbootstrap.com/). The correlation-based network module was developed by open-source graph network library Cytoscape.js 3.15.1 (https://js.cytoscape.org/). The draggable widget of workflow is developed based on a modified version of diagram (topology, UML) framework Le5le 0.3.11 (https://github.com/le5le-com/topology). The entire software was hosted on an Ali server with 64GB of RAM and 16 virtual CPUs with 3.2 GHz each. iMAP supports multi-user multi-threading operation and can be used by several users at the same time. The backend R scripts are available at https://github.com/IP4M/R-scripts-for-iMAP.

## Applications

### Omics Data Sets

Two independent data sets were utilized to demonstrate the metabolomics data analysis performed by iMAP. Workflow with the predictive model building was performed on 234 clinical samples (Zhu et al., 2014) from three groups (66 colorectal cancer patients, 76 polyp patients, and 92 healthy controls). The data set contains 113 metabolites detected by targeted serum metabolic profiling. Gender and age were matched in each group, and no statistical significance was found by the Mann-Whitney U test. A correlation-based network was constructed from proteomics and metabolomics data of 308 clinical stool samples (Lloyd-Price et al., 2019) from patients with inflammatory bowel disease and health control in the IBD database at https://ibdmdb.org/.

### Predictive Model Building

**Figure 4:**
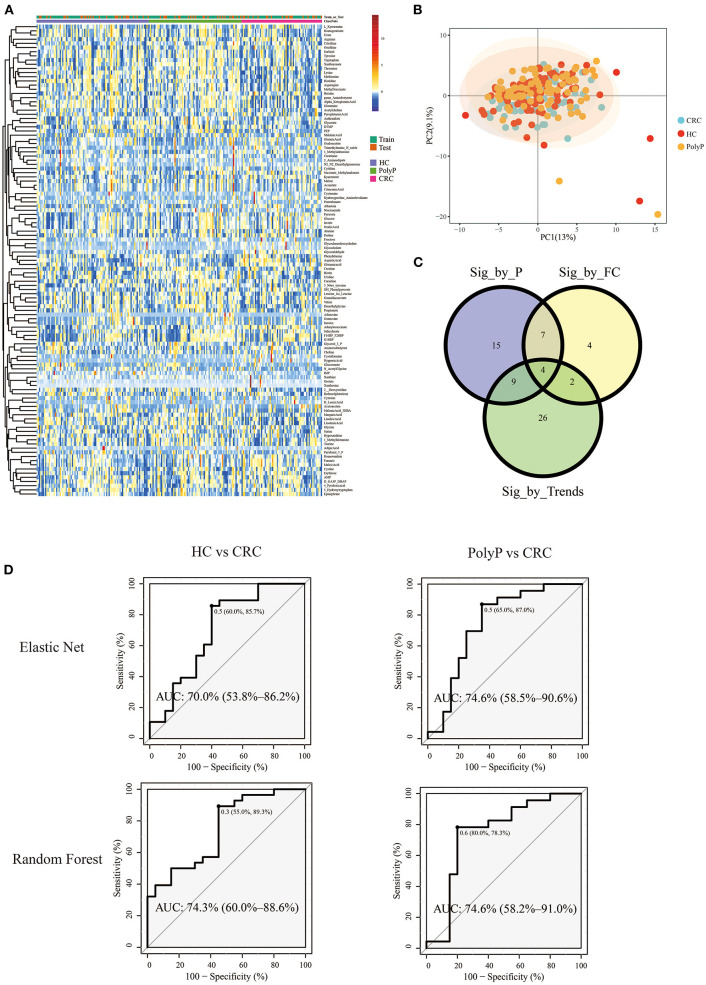
Metabolomic profile and predictive models built by selected metabolites. **(A)** Heatmap showed the Z-score value of metabolites intensity. The annotation bars above the heatmap showed groups and train/test information of the samples. **(B)** PCA scores plot. **(C)** Venn plot showed the intersections of metabolites filtered by three different criteria. **(D)** ROC plot of random forest and elastic net. The optimal cutoff value, specificity, and sensitivity were shown beside the optimal cutoff point in the ROC plot. The AUC and 95% Confidence interval was shown in the middle of the ROC plot.

Pipeline and pipeline were performed separately to illustrate the difference between different model building pipelines. Samples were divided into the train set and test set (train: test = 8:2) by stratified random sampling. The intensity of metabolites in train and test samples was shown in the heatmap (Figure 4A). PCA scores plot (Figure 4B) with the first two principal components shows that principal component scores among three groups were gathered very close, and most areas of the confidence ellipse from the three groups were overlapped to each other. Train set data were used for metabolites-selection and modeling. Metabolites that met all three following criteria were selected for predictive model building:

(1) Pairwise *post-hoc P* < 0.05 between ≥1 group pair by Tukey test after Kruskal Wallis test.
(2) |log_2_FC| > 0.25 between colorectal cancer (CRC) patients and polyp patients (PolyP), and |log_2_FC| > 0.25 between CRC patients and healthy controls (HC).

Detailed metabolites-selection results and model building were summarized in Supplementary Tables 1–8 separately (Screenshot of the workflow and parameters used were shown in Supplementary Figure 3). Venn plot (Figure 4C) showed that 4 metabolites, including Adenylosuccinate, Histidine, Hydroxyproline Aminolevulinate, and Linolenic Acid, meet all the criteria in selection. Random forest (RF) modeling and elastic net modeling with these 4 selected metabolites were constructed. The train set (Pipeline) and test set (Pipeline) were used to validate the model separately. Model AUC in the train set and test set were shown in Table 2, and the ROC plot between CRC-PolyP and between CRC-HC are shown in Figure 4D. AUC in the train set (Pipeline) is higher than AUC in the test set (Pipeline) in all conditions except elastic net model between group HC and group PolyP. AUC in the train set even reached 1 in the RF model between all group pairs. However, the error rates of out-of-bag (OOB) samples was 0.364, 0.255, and 0.453, respectively, between CRC-PolyP, CRC-HC, and PolyP-HC (Supplementary Figure 3). The high OOB error rates (especially for PolyP-HC) indicated that the AUC of a model from Pipeline could be overly optimistic and had a higher risk of overfitting. Additionally, the AUC values of all the test sets were <0.8, which verified that Pipeline was overly optimistic and had a higher risk of overfitting.

**Table 2:** AUC of ROC in the test set and train set for pair group discrimination.

**Group pair**	**Method note**	**AUC in test^a^**	**AUC in train^b^**
HC vs. CRC	Random forest	0.743	1
HC vs. CRC	Elastic net	0.7	0.865
CRC vs. PolyP	Random forest	0.746	1
CRC vs. PolyP	Elastic net	0.746	0.823
HC vs. PolyP	Random forest	0.528	1
HC vs. PolyP	Elastic net	0.5	0.5

a *AUC by pipeline, model was built by the train set and validated by the test set*.

b *AUC by pipeline, model was built by the train set and validated by itself*.

### Correlation-Based Network

Both proteomics data and metabolomics data were processed separately in the data-processing model. Samples with > 90% zeros and variables with > 40% zeros were removed. Moreover, the data in each sample were normalized to counts per million. Two hundred and two shared samples in two data sets were reserved, and 234 metabolites and 112 proteins were reserved after processing (Supplementary Tables 9, 10). Spearman correlation analysis was performed on the processed data. FDR < 0.05, and |*r*| > 0.3 were used to filter correlations between metabolites and proteins (Supplementary Figure 5). The 95 correlations qualified to the threshold were selected as edges to construct the correlation-based network (Supplementary Tables 11, 12). The correlation-based network contains 33 proteins, including glutamate dehydrogenase [NAD(P)(+)], 6-phosphofructokinase, and aspartate-semialdehyde dehydrogenase, and contains 39 metabolites such as adipate, 3-methyladipate/pimelate, and pentadecanoate.

**Figure 5:**
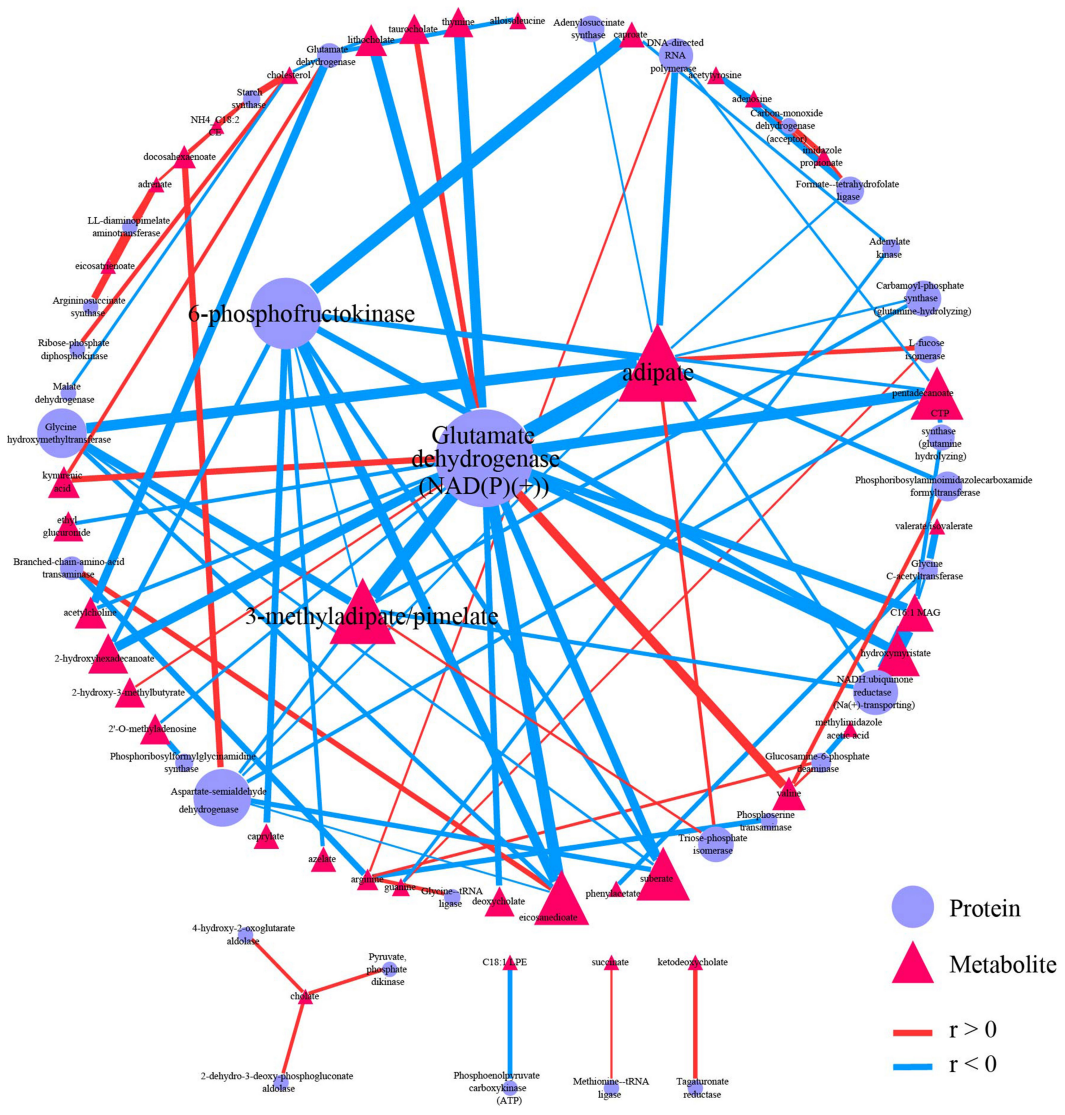
Correlation-based network. Network showed 95 qualified (|*r*| > 0.3 and FDR < 0.05) correlation. Nodes were colored and shaped by variables type (metabolite or protein). Nodes size depends on the evcent centralities of the variables. Edges were colored by the sign of r, and edges' width depends on |*r*|. A primary network plot was generated in iMAP, and the layout of nodes was modified in Cytoscape 3.8.0. A circular layout was applied, and the four biggest nodes were placed in the center manually.

Graph-theory centralities were calculated for nodes in the network, and the network was visualized by the correlation-based network module based on Cytoscape.js. A primary network plot was constructed based on regular visual settings, including mapping different colors and shapes to nodes from different data sets, mapping node size to evcent centrality, mapping edge color and width to r. Users can import the primary network into Cytoscape and modify it directly. Users can also import the edge table and the node table into Cytoscape for network construction (Supplementary Tables 12, 13). After modifying the layout and edge width, the final network plot was shown in Figure 5. Glutamate dehydrogenase [NAD(P)(+)] was the biggest node in proteins, followed by 6-phosphofructokinase. Moreover, these two biggest metabolites have wider edges with metabolites than others in the network. Adipate and 3-methyladipate/pimelate were the biggest two nodes in metabolites. The two biggest protein nodes are significantly correlated to the two biggest metabolite nodes in the network, indicating that these four molecules may be critical hubs for the network and may play essential roles in IBD progression.

## Function Comparisons With Existing Tools

Functions of iMAP and 8 widely used integrated platforms were compared in Table 3. All platforms can preprocess MS and MS/MS data except iMAP and Metabox. iMAP and W4M have more data processing methods than the other platforms. iMAP, IP4M, and MetaboAnalyst have more metabolite selection methods than the other platforms. Only the iMAP, IP4M, and MetaboAnalyst can build predictive models. MetaboAnalyst needs to select samples for model building and validation manually. In iMAP, this step can be done automatically by the “Train set ratio” defined by user. iMAP is the only platform provides 3 types of usage modes, predefined workflows, independent modules, and customized workflows, while the other platforms support one or two modes. However, some commonly used methods, such as linear regression with covariant variables, cross-validation in predictive modeling, and DESeq2, are not supported in iMAP. Few functions are provided for time series analysis, function-based network analysis, and multi-omics data integrative analysis. Overall, iMAP is a good alternative tool with complementary functions to existing metabolomics data analysis platforms.

**Table 3:** Function comparisons of iMAP and other 7 tools.

**Function tool**	**iMAP**	**IP4M**	**MetaboAnalyst 4.0**	**W4M**	**Galaxy-M**	**XCMS-online**	**MZmine2**	**MetaBox**	**MS-DIAL**
MS and MS/MS data process	–	√	√	√	√	√	√	–	√
**Data processing**									
Normalization, scaling, and zero filling	√	√	√	√	√	√	√	√	√
Transformation	√	√	√	√	√	–	–	√	√
Variables merging	√	√	–	√	–	–	√	–	–
Remove variables or samples	√	√	√	√	√	–	√	–	√
batch-effect correction	√	–	√	√	–	√	–	√	√
**Metabolites-selection**									
Univariate analysis	√	√	√	√	√	√	√	√	√
PCA/(O) PLSDA	√	√	√	√	√	√	√	√	√
Regression analysis	√	√	–	–	–	–	√	–	–
Time series analysis	–	–	√	–	–	–	–	–	–
SVM	√	√	√	–	–	–	–	–	–
RF	√	√	√	–	–	–	–	–	–
Correlation/distance	√	√	√	√	–	–	√	√	√
Gradient boosting (GB)	√	√	–	–	–	–	–	–	–
Pathway enrichment and topology analysis	√	√	√	–	–	√	–	√	√
**Other functions**									
Predictive model building	√	√	√	–	–	–	–	–	–
Hierarchical cluster	√	√	√	√	–	√	√	√	√
Plotting tools	√	√	√	√	–	√	√	√	–
Omics data integration analysis	–	√	√	–	–	√	–	√	–
Integrated workflows	√	√	√	√	√	√	√	√	–
customized workflow	√	–	–	–	–	–	–	–	–
Developing language	R, Java	R, Java, Perl	R, Java	R, tabular	R, Python, Matlab	R	R, Java	R,	C#
Platform	Web server	Local GUI; Windows/Linux/Mac OS	Web server; Local R package	Web server	Web server	Web server	Local GUI; Windows/Linux/Mac OS	Local GUI; Windows/Linux/Unix;Local R package	Local GUI; Windows

## Discussions

In the first application on real world data, 4 biomarkers selected by a combination of 3 criteria in iMAP were also listed as biomarkers by original researchers (Zhu et al., 2014). Combining adenylosuccinate, histidine, hydroxyproline/aminolevulinate, and linolenic acid, predictive model ROC between group CRC and the other two groups can reach an AUC > 0.70. Significant expression change of adenylosuccinate in CRC has been reported (Brown et al., 2016). Adenylosuccinate lyase (ADSL) converts adenylosuccinate to AMP and fumarate, which is an essential part of the purine nucleotide cycle. ADSL was reported to be upregulated in various malignancies, including CRC. ADSL upregulation can upregulate fumarate expression and may enhance cell proliferation, migration, and invasion through regulation of killer cell lectin-like receptor C3 (Park et al., 2018). Isozyme shifted of adenylosuccinate synthase in rat and human neoplasms has also been reported (Ikegami et al., 1989). Downregulation of histidine in CRC was observed in the train data set and several other studies (Masini et al., 2005; Wierzbicki et al., 2009; Qiu et al., 2010; Nishiumi et al., 2012). The downregulation of histidine may be due to the acceleration of decarboxylation from histidine to histamine in CRC patients, caused by the increased activity of histidine decarboxylase. Hydroxyproline is abundant in collagen, and interstitial collagen is a major constituent of the CRC matrix. This may explain the significant change of hydroxyproline observed in CRC patients (Karna et al., 2012; Holowatyj et al., 2020). Aminolevulinate (also known as 5-aminolevulinic acid or 5ALA) is a precursor to fluorescent protoporphyrin IX. Moreover, the regulatory enzyme activity for these processes has a significant increase in the CRC tissue. Thus oral administration of 5ALA and observe the tumor-specific accumulation of fluorescence from protoporphyrin IX can differentiate CRC tissue and normal tissue, which has already been clinically used to detect the peritoneal dissemination of CRC (Kondo et al., 2014). Linolenic acid was reported significantly changed in adipose tissue from newly diagnosed CRC patients compared with control subjects (Cottet et al., 2015). EFSA recommends daily intakes of linolenic acid for 4% of total energy since 2010, and the protective effect of linolenic acid from CRC risk (OR = 1.20, 95% CI, 1.07–1.36) is observed in a case-control study with 1953 CRC patients and 4,154 controls (Turati et al., 2012). The abundant evidence from studies for all 4 biomarkers selected by iMAP shows that by merely inducing trends among groups into metabolites-selection, users can improve the result's interpretability. Thus, different research types (such as exploratory study and biomarkers identification study) may require different selection strategies. Additionally, metabolites-selection strategies may also need to be adjusted based on the data. In this demonstration, neither PLS-DA nor OPLS-DA model was performed because the samples from three groups can hardly differentiate from other groups in the principal component score plot. Additionally, there was no obvious patterns among groups in the heatmap plot (Figure 4A). However, biologically significant biomarkers could be discovered, and an acceptable model with AUC > 0.7 could be built via the customized workflow. These conditions may also happen to data from other users, which is another reason for the customized workflow being helpful in metabolomics data analysis. Gaps between AUC in the train set and AUC in the test set showed that Pipeline could be overly optimistic and have a higher risk of overfitting. Five methods, including RF, Gradient boosting model, Logistic regression, SVM, and Elastic Net model, are available for predictive modeling in iMAP at present. RF is one of the frequently used machine learning methods in lab and industry and may be over-fitting sometimes. The Elastic Net is a regularized regression method that linearly combines the L1 and L2 penalties of the lasso and ridge methods to combating the over-fitting. In our work on the demonstration data. RF model in the train data was over-fitted with out-of-bag (OOB) error rates of 0.364, 0.255, and 0.453 between CRC-PolyP, CRC-HC, and PolyP-HC, respectively. AUC in train set get by Elastic Net was close to AUC in the test set than that get by RF model. And AUC in the train set [Pipeline] is higher than AUC in test set [Pipeline]. We compared RF and Elastic Net to illustrate:

(1) Predictive models may be over-fitting sometimes (especially those built by Pipeline).

(2) Models with L1 and L2 regularization might be helpful for users to combating over-fitting.

(3) Pipeline [and Pipeline] can also help users to combat over-fitting.

There are some reasons for the not good result in distinguishing HC and PloyP by metabolic features in the predictive models, including the high prevalence in old people (Pan et al., 2020; Rex et al., 2020), only part of polyp patients have neoplastic polyps (Lieberman et al., 2008) and the neoplastic polyps may take decades to progress (Rutter et al., 2015; Short et al., 2015), and most low-risk polyps may have limited influence on the serum metabolism (Wang et al., 2018).

Network plots can directly and effectively summarize the information from correlation analysis cause the importance of variables can be emphasized by node size, and correlation strength between variables can be showed by edge width. Readers can quickly locate critical hubs by these visual features in the network. Glutamate dehydrogenase [NAD(P)(+)], 6-phosphofructokinase, Adipate, and 3-methyladipate/pimelate were critical hubs in the demonstration network plot. The connection between these hubs and IBD have already been reported except 6-phosphofructokinase. Glutamate dehydrogenase enzyme immunoassay (EIA) is a commonly used rapid testing option for identifying Clostridium difficile infection (CDI). CDI has a higher prevalence in IBD patients, and the prevalence has been increasing in recent years (Sinh et al., 2011; Albarrak et al., 2019). The influence on inflammation induced by NAD-dependent enzymes, including Sirtuin 1 (Caruso et al., 2014; Wellman et al., 2017; Sedda et al., 2018) and NAMPT (Gerner et al., 2018) may interpret the relation between glutamate dehydrogenase [NAD(P)(+)] and IDB. Adipate was reported to be significantly elevated in urine from rats with IDB (Wang et al., 2020). 3-methyladipate was reported to be significantly decreased in IDB patients in an Italian cohort study (Santoru et al., 2017). 3-methyladipate was also the only metabolites in the study to show a significant correlation with *Akkermansia muciniphila* species, which was found to be selectively decreased in the fecal microbiota of patients with IBD (Plovier et al., 2017). Pimelate was reported to be significantly elevated in urine from children with IDB (Martin et al., 2017). Moreover, adipate and 3-methyladipate/pimelate were also emphasized as critical hubs in the correlation-based network between microbiome data and metabolome data in the original report. No connection between 6-phosphofructokinase and IBD was reported yet. Thus, it may be study-worthy by “data-driven strategy” because 6-phosphofructokinase has the second evcent centrality in proteins and significantly correlates with the other two critical hubs in metabolites.

The interpretability of key hubs indicates that the node centrality can help locate the disease process's critical entities. To better understand the complicated disease process, multi-omic methods are increasingly needed. A correlation-based network is a powerful tool for analyzing and interpreting the multi-omic result. Using the correlation-based network module in iMAP, users can construct and analyze network plots to demonstrate their multi-omics result conveniently.

## Conclusions

This study reported a new web tool, iMAP, for metabolomics data analysis. As the updated version of IP4M, besides enhanced visualizations and results, more methods and modules, and various predefined workflows, iMAP enables users to construct customized workflows taking into considerations of their specific study aims and data characteristics. Most modules in iMAP can also be utilized on other omics data sets. Although there are still many limitations, iMAP is an alternative tool with complementary powers to existing metabolomics data analysis platforms.

## Data Availability Statement

The original contributions presented in the study are included in the article/Supplementary Material, further inquiries can be directed to the corresponding author/s.

## Author Contributions

JL, TC, and DZ: conceptualization and supervision. DZ, WZ, and TS: design and developed software. YW, YC, and TS: data curation. DZ, YW, YC, and WZ: formal analysis. TC: funding acquisition. TC, DZ, YC, YW, TS, and WZ: writing—original draft. TC, JL, DZ, YW, YC, and TS: writing—review & editing. All authors test and provide feedback.

## Conflict of Interest

DZ, WZ, YW, YC, and JL were employed by the company Metabo-Profile Biotechnology (Shanghai) Co. Ltd. The remaining authors declare that the research was conducted in the absence of any commercial or financial relationships that could be construed as a potential conflict of interest. The reviewer RW declared a past co-authorship with one of the authors TC.
