# Supplementary material for: iMAP: A Web Server for Metabolomics Data Integrative Analysis
Source: Front Chem. 2021 May 5;9:659656. doi: 10.3389/fchem.2021.659656 (PMC8133432; doi:10.3389/fchem.2021.659656)
Supplement: Supplementary file 1 [file Data_Sheet_1.PDF]

Figure S1A. Access to the predefined data-processing and workflow demos.

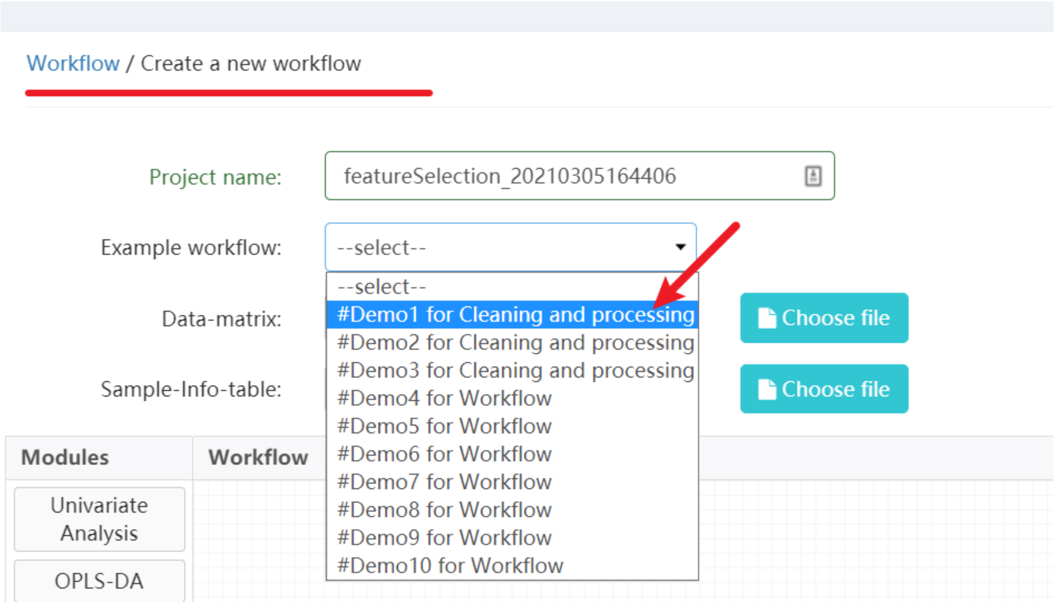

Figure S1B. Demo1 for Cleaning and processing with the parameters utilized.

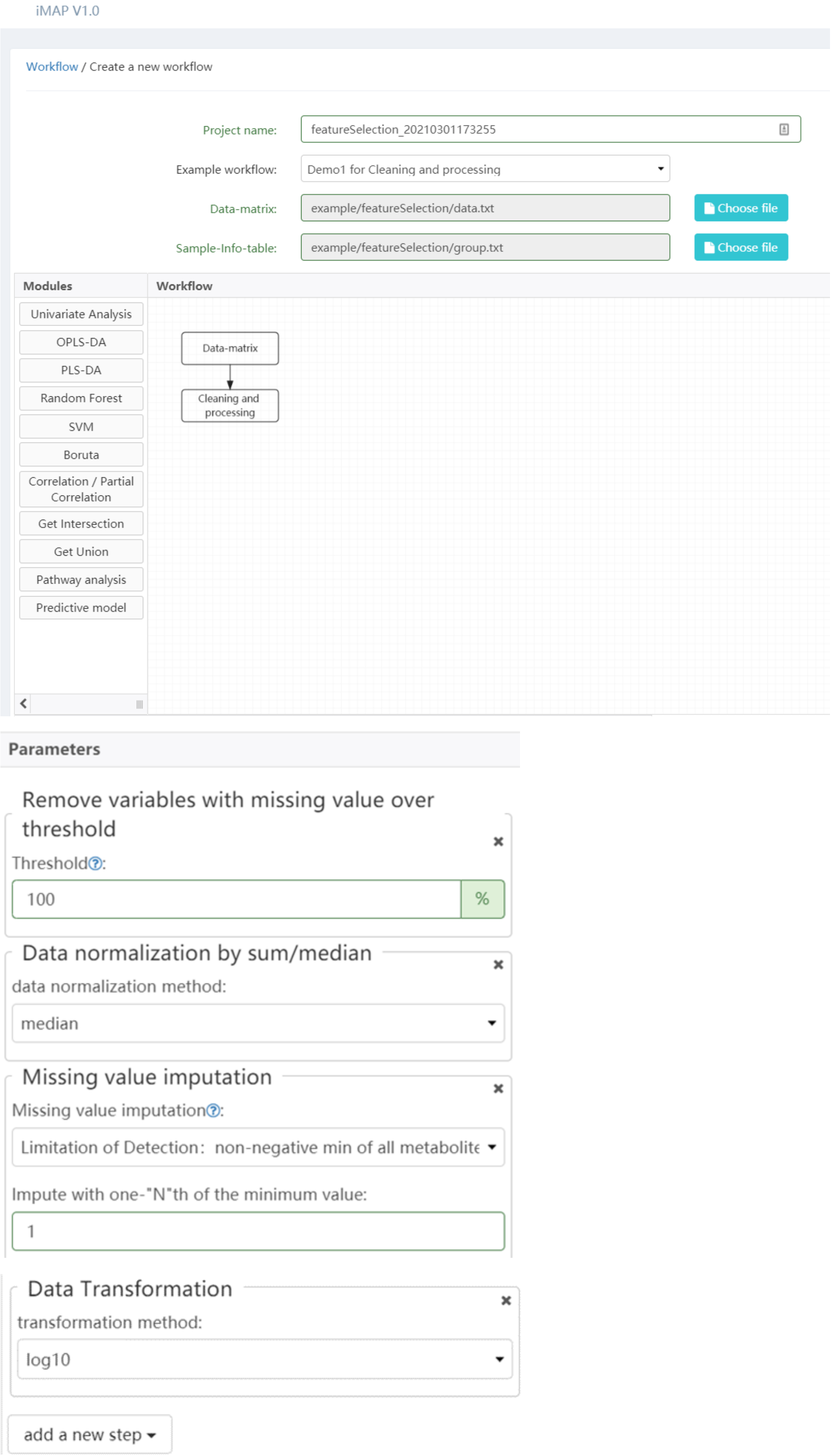

Figure S1C. Parameters of Demo2 for Cleaning and processing.

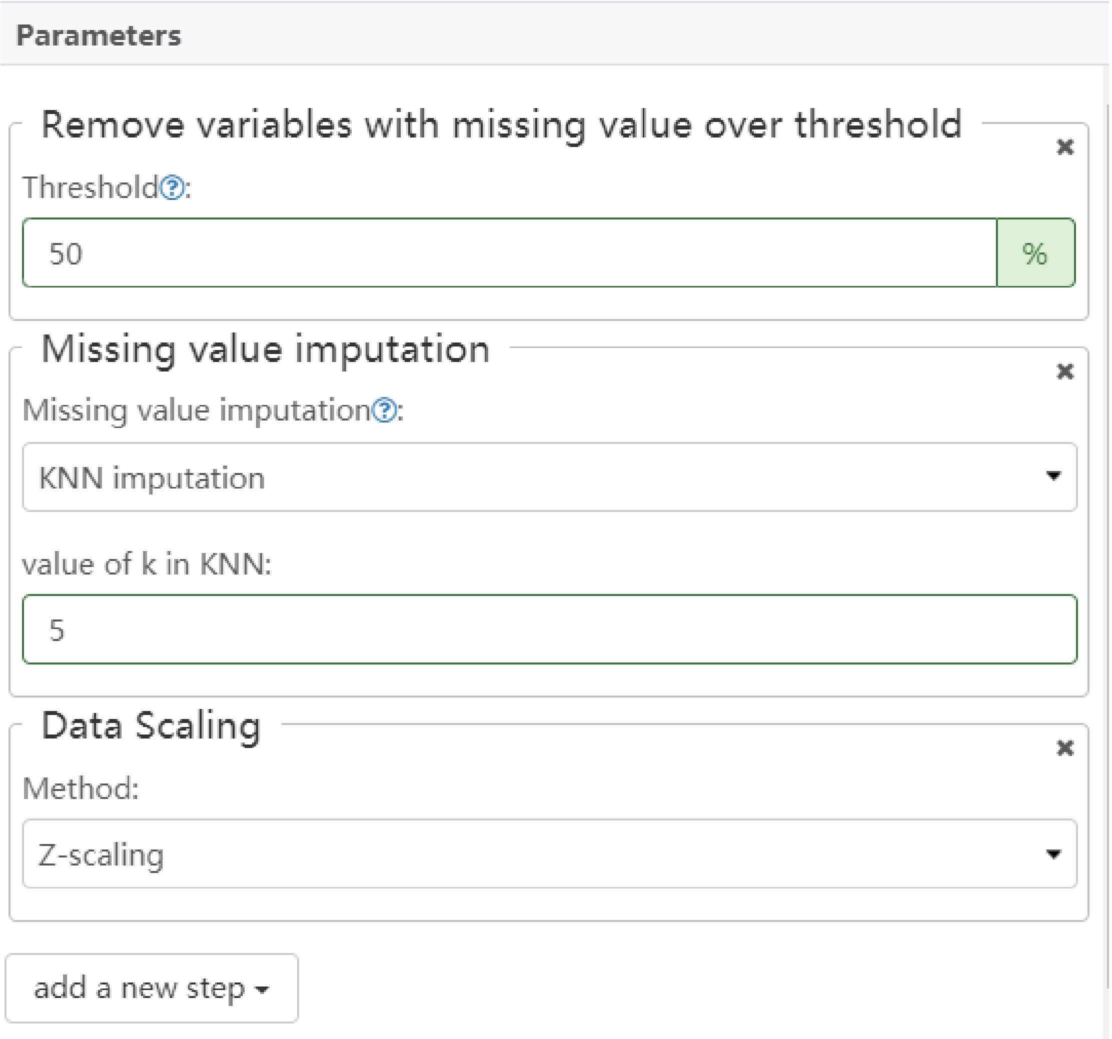

Figure S1D. Parameters of Demo3 for Cleaning and processing.

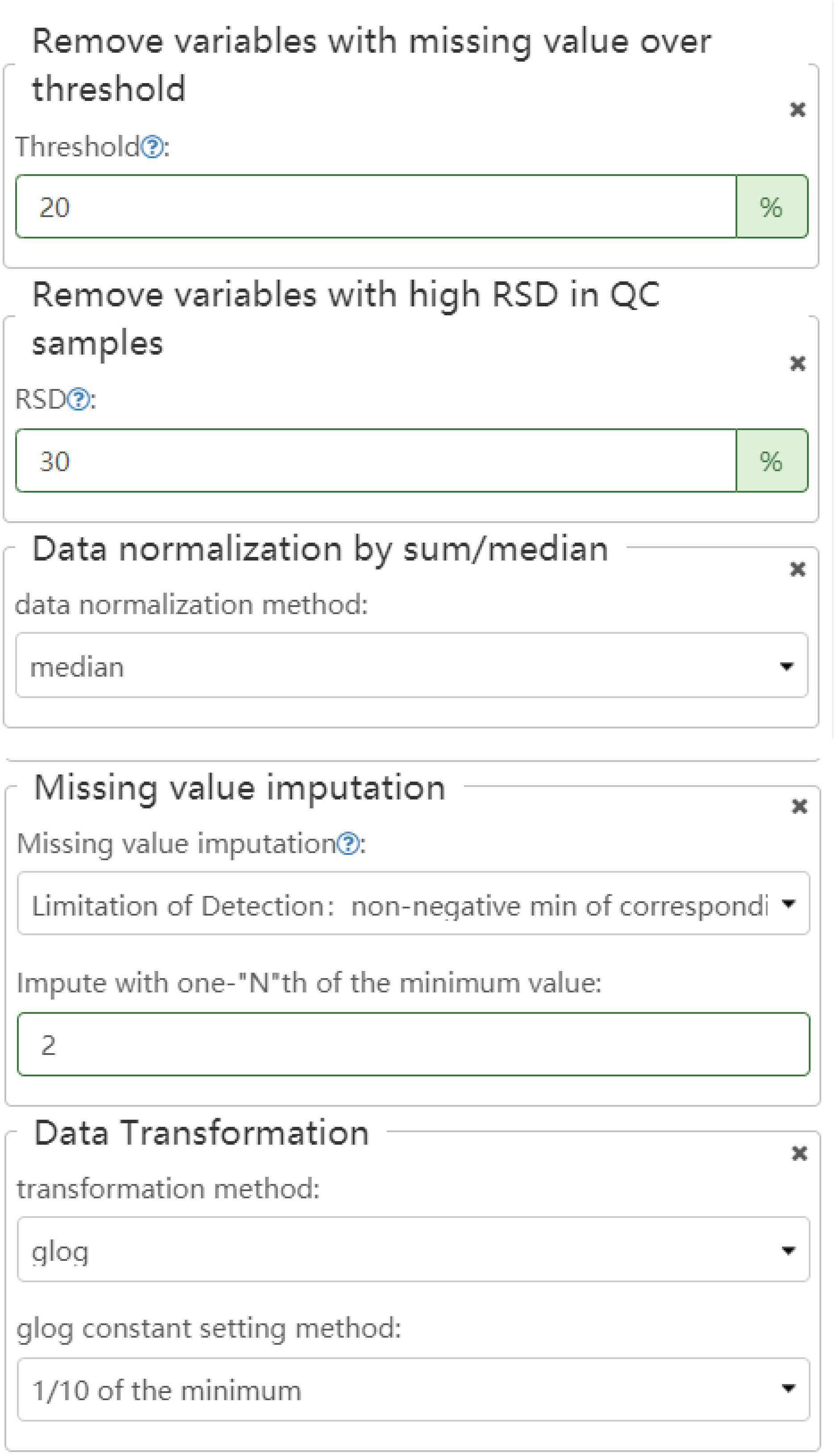

Figure S1E. Demo4 for workflow.

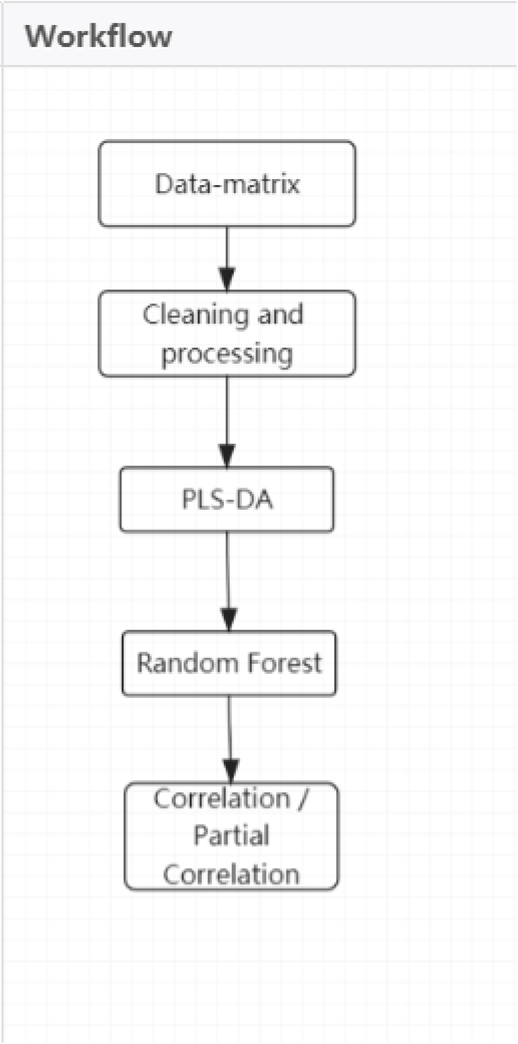

Figure S1F. Demo5 for workflow.

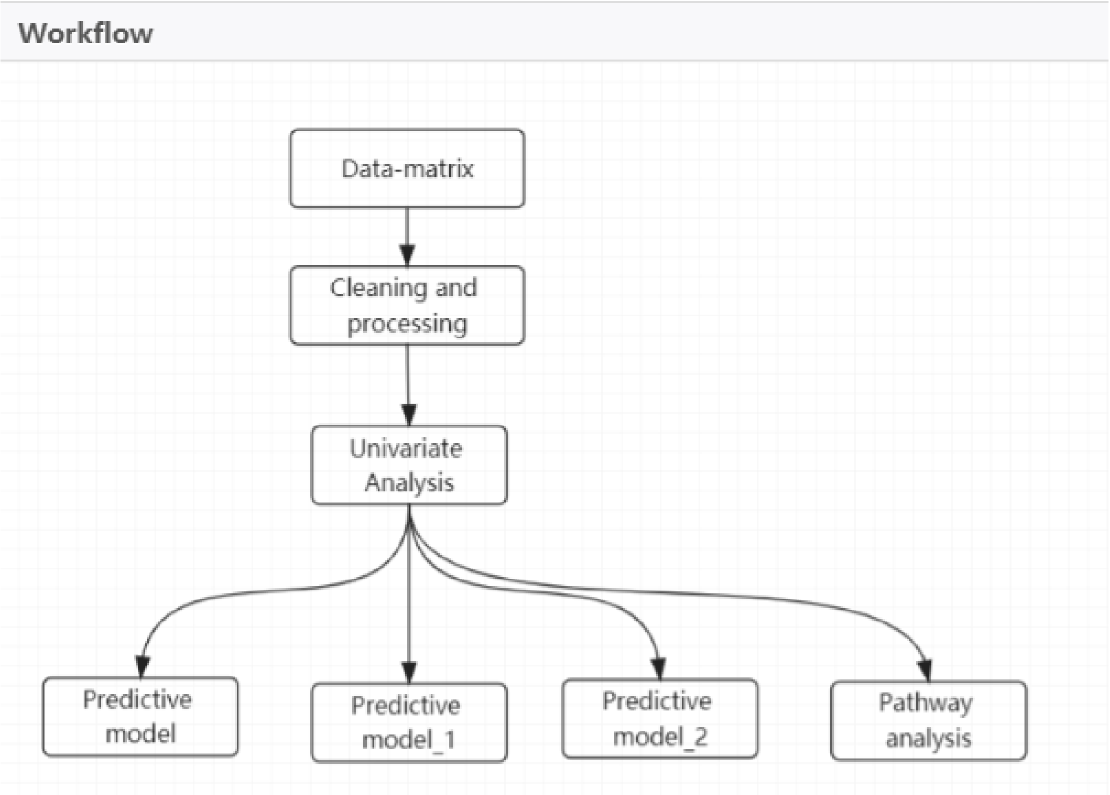

Figure S1G. Demo5 for workflow (Method, P-value cutoff, and FDR cutoff in Univariate Analysis module).

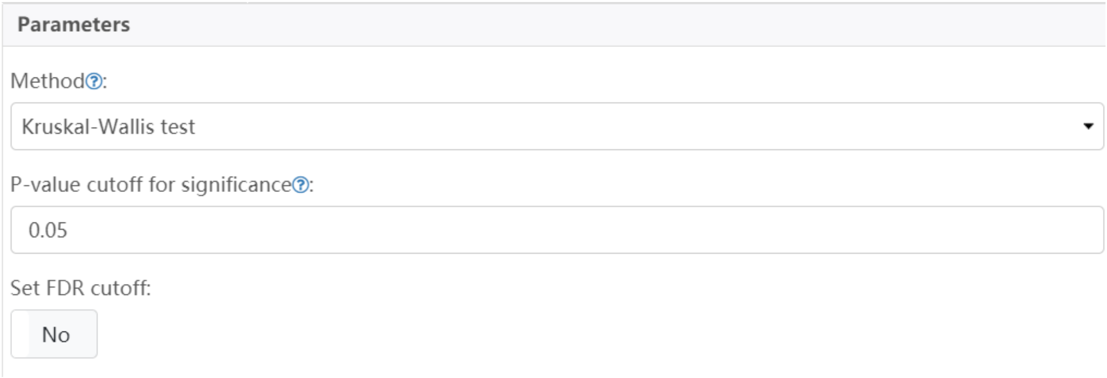

Figure S1H. Demo5 for workflow (combination of post-hoc P-value, log2FC between group pairs in Univariate Analysis module).

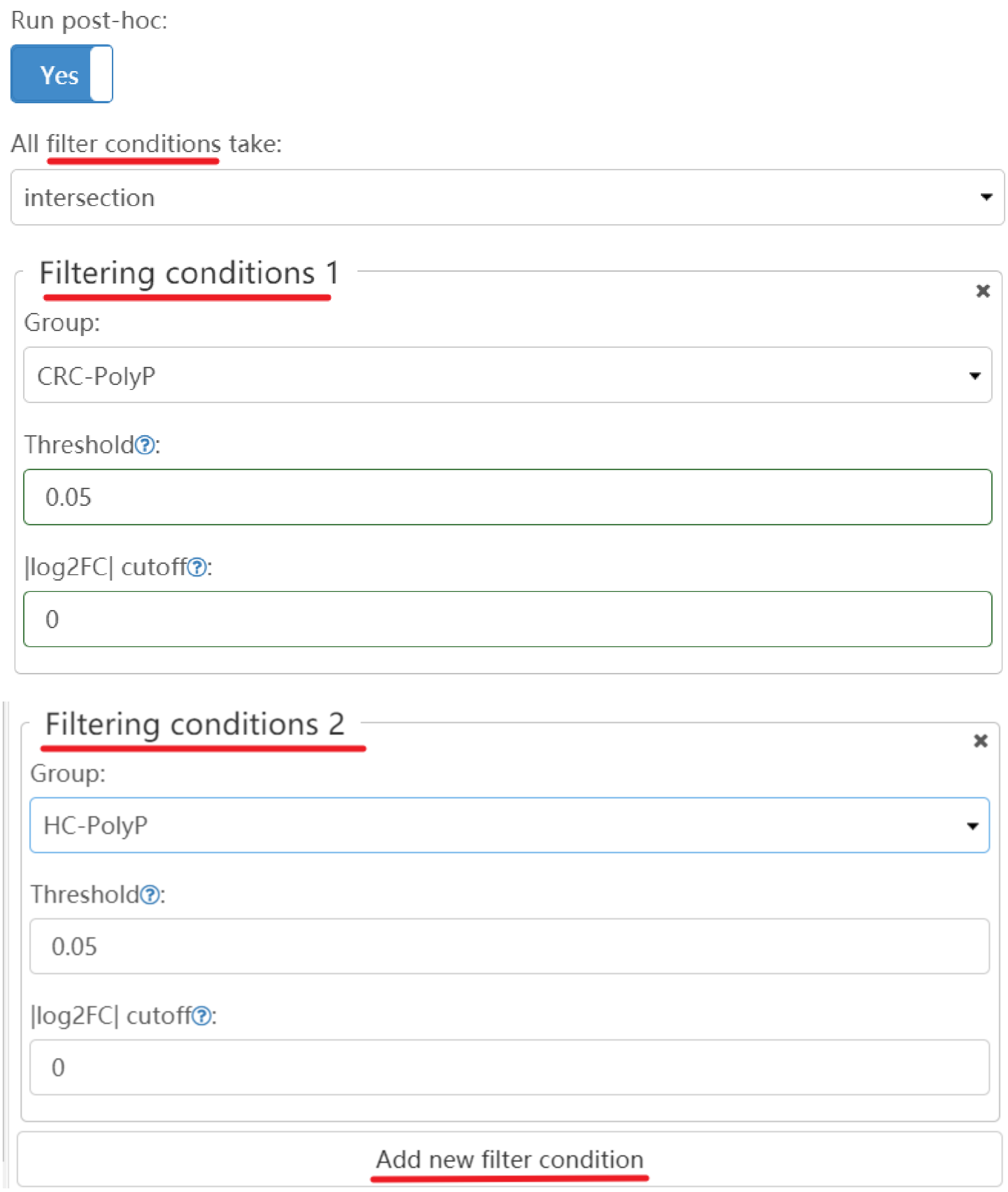

Figure S1I. Demo5 for workflow (trends-among-groups in Univariate Analysis module).

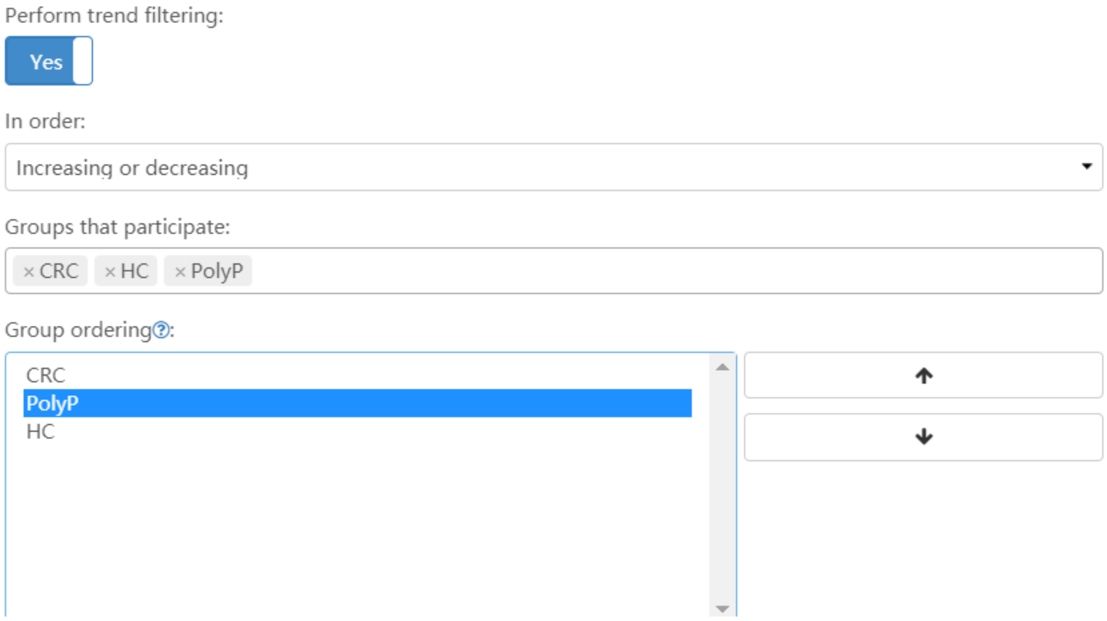

Figure S1J. Demo5 for workflow (parameters in the predictive model module).

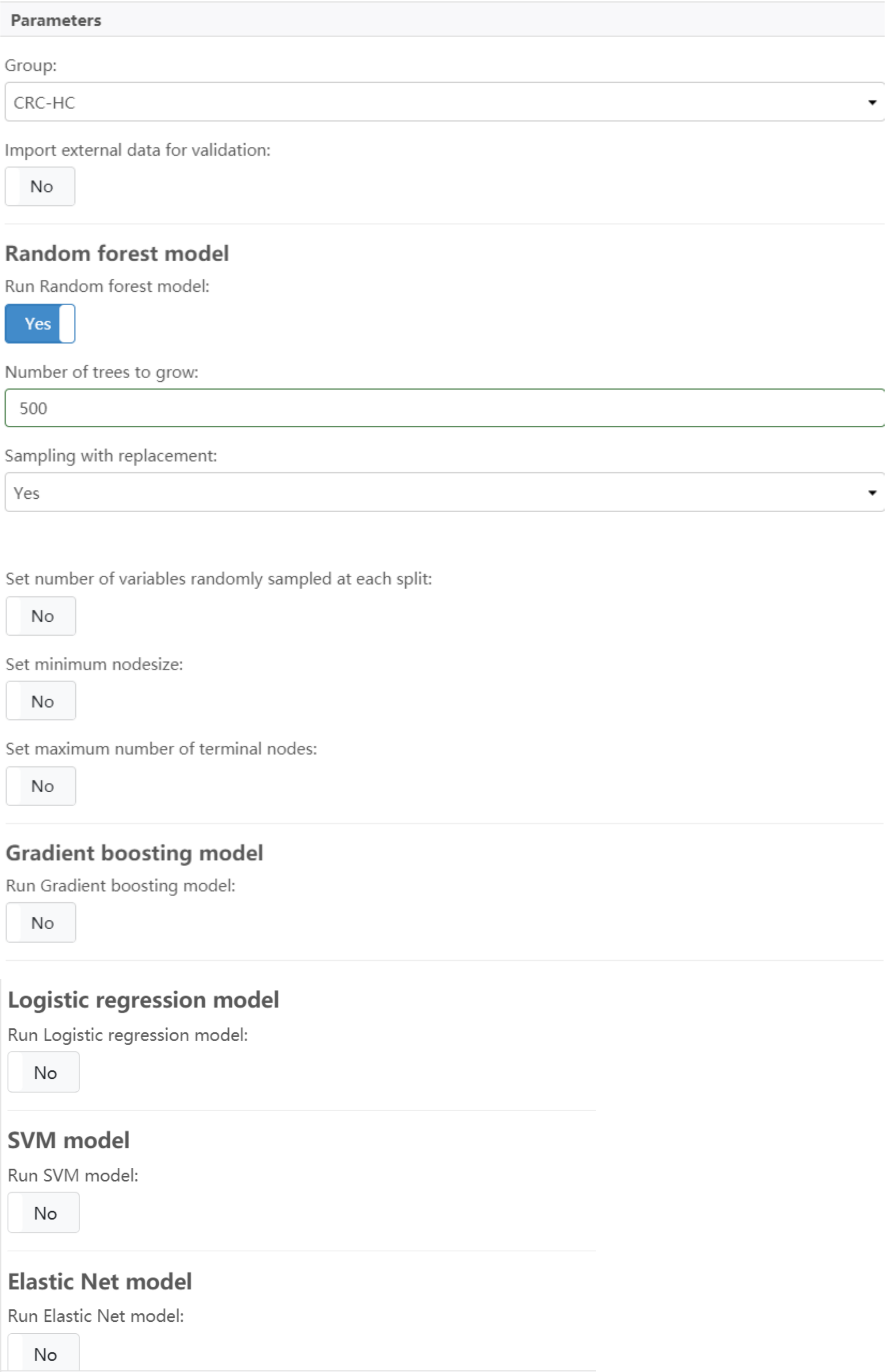

Figure S1K. The parameter to choose group pairs in post-hoc P-value and predictive modeling.

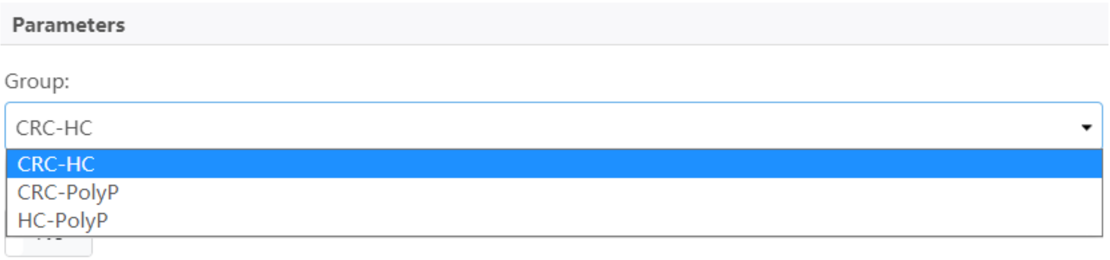

Figure S1 Screenshot about data-processing and workflow demos in iMAP. Ten example workflows for data processing and analyzing, summarized from published metabolomic studies, were provided at present. Screenshots of demo1-5 were shown and introduced here, and detailed information for all example workflows was provided in section ”Example workflows” in https://imap.metaboprofile.cloud/metaboCloundPlatform/user/guideBefore. **(A)** Access to choose predefined data-processing and workflow demos. **(B)** After choose demo1, users can see the workflow in the Workflow-panel and check the parameters used in the Parameters-panel. Data processing demo1 was summarized from a multi-omic study (Bushman et al., 2020) about IBD. Variables with 100% missing values were removed, and data were normalized by the median of each variable. Missing values were imputed by the minimum value across all samples (impute missing value according to the limit-of-detection, LOD), and Log10 transformation was performed. **(C)** Parameters of demo2 for Cleaning and processing (Wozniak et al., 2020). Samples with ⩾ 50% missing value were removed, and missing values were imputed by KNN. The data were scaled into Z-Score for the following analysis. **(D)** Parameters of demo3 are based on three studies. Remove variables with a over 20% missing value rate (‘80% rule’) comes from a guildeline for mass spectrometry-based metabolomics data (Smilde et al., 2005). Remove variables with > 30% RSD in QC samples comes from another protocol guideline (Want et al., 2010). Other parameters come from a biomarker screening study (Sreekumar et al., 2009). **(E)** Demo4 for workflow(Bowerman et al., 2020) was summarized from a metabolomics biomarker study in chronic obstructive pulmonary disease (COPD) patients. Data were normalized by the median. Missing values were imputed by the minimum of each compound, and the data were ln transformed. After cleaning and processing, metabolites were pre-selected by PLS-DA VIP, and then selected by the Random Forest. After two selection steps, correlation analysis were performed between the final selected metabolites and other data set. **(F)** Demo5 for workflow(Sreekumar et al., 2009). This demo shows that multiple predictive modules (with different parameters inside and different suffixes) can be integrated into a workflow. Other modules can also be multiple to construct the flexible workflow. **(G-I)** Parameters in Univariate Analysis module in demo5. **(G)** Comparing method, P-value cutoff for significance, and FDR cutoff in Univariate Analysis module. **(H)** Filter conditions can be combined to select metabolites. Tukey post-hoc P-value (parameter Threshold in the screenshot) and log2FC between chosen group pairs can be used to select qualified metabolites. Each condition will select metabolites that fit all criteria inside the condition, and a union or intersection set can be integrated from all conditions. Users can also add more conditions by clicking the “Add new filter condition” button. **(I)** Trends-among-groups in Univariate Analysis module. Users can choose groups to be participated in the trends setting and change the order of the participated groups. Note: In the original study: Health-Control->Localized-Cancer (Early-Stage)->Metastasis-Cancer (Late-Stage)

was used in the trend. And in the example workflow: Health-Control (HC)->PolyP (precancerous)->CRC (Colorectal-Cancer) was used instead. **(J)** Parameters in predictive model module. Five methods for predictive modeling were available, and RF was chosen in the demo. **(K)** The parameter to choose group pairs in filter conditions and predictive modeling. Some analysis steps can process multi-group data (have more than two groups), while others can only process two group data once a time (such as OPLS-DA, Fold Change calculation, and predictive modeling). To integrate these analyses into one workflow, we added the "Group" parameter to pick up two groups each time for those analysis steps like the predictive model. Note, some methods not accessible in iMAP were replaced by similar methods, such as sPLS-DA were replaced by PLS-DA in the Demo4. Example workflow 4-10 showed different analysis strategies to select metabolites and build a predictive model. One example workflow builds the predictive model by all detected metabolites directly without any metabolite selection (Bushman et al., 2020). Some example workflows select metabolites by machine-learning method or statistically-significance and build a predictive model based on the selected metabolites (Sreekumar et al., 2009; Liu et al., 2017; Xuan et al., 2020; Adam et al., 2021). Some other example workflows perform a pre-filter selection and then select metabolites from the pre-filtered metabolites (Bowerman et al., 2020; Wozniak et al., 2020; Chen et al., 2021). Additionally, some example workflows select the metabolites by combining multiple methods (Liu et al., 2017; Liu et al., 2019; Oh et al., 2020; Chen et al., 2021) or compare between different group pairs (Xuan et al., 2020; Liu et al., 2021), and a union/intersection of the selected metabolites will be used for the following analysis steps, such as pathway enrichment analysis or predictive model building. Besides these seven predefined workflows summarized from the metabolomic studies mentioned above, users can construct their customized workflows based on these example workflows and rerun the new workflow with their data. These example workflows were provided to show that various analysis strategies can be utilized for metabolomic data mining. Users can construct their workflow according to their data and study design.

Figure S2A. Example input data in the workflow.

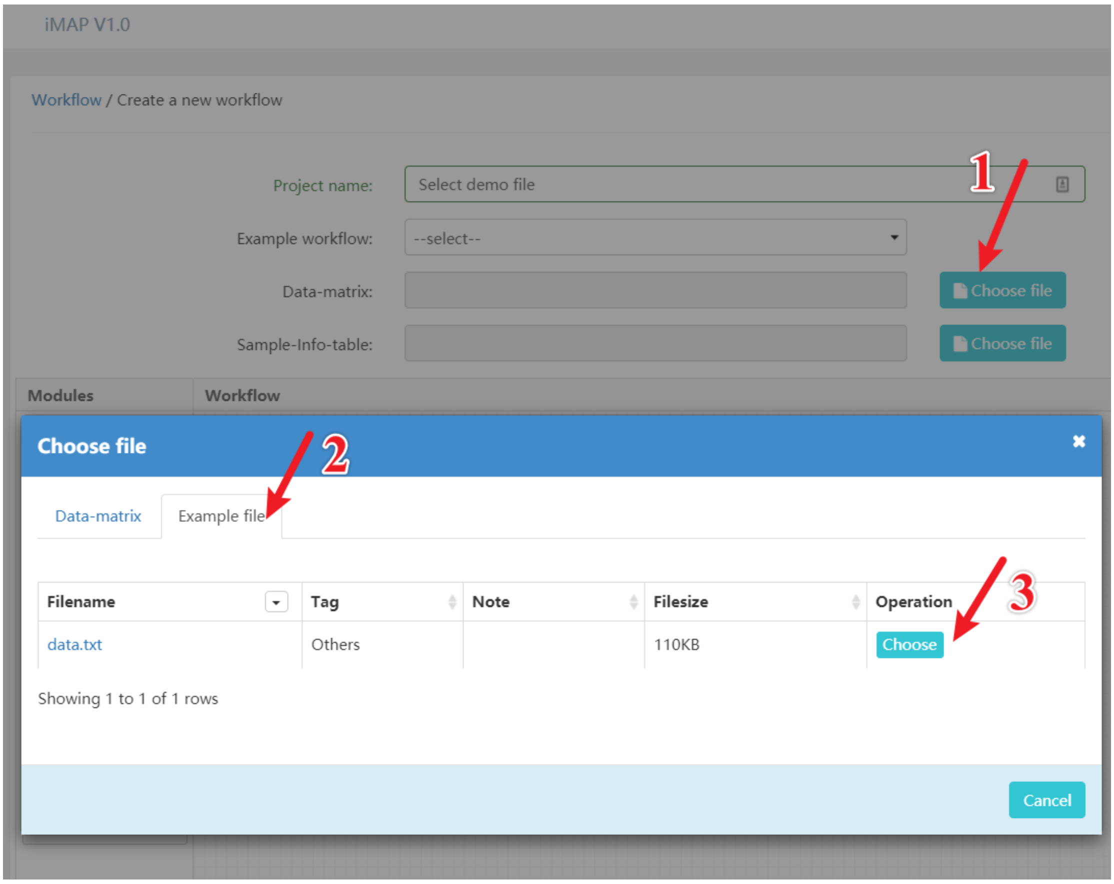

Figure S2B. Example input data in each module.

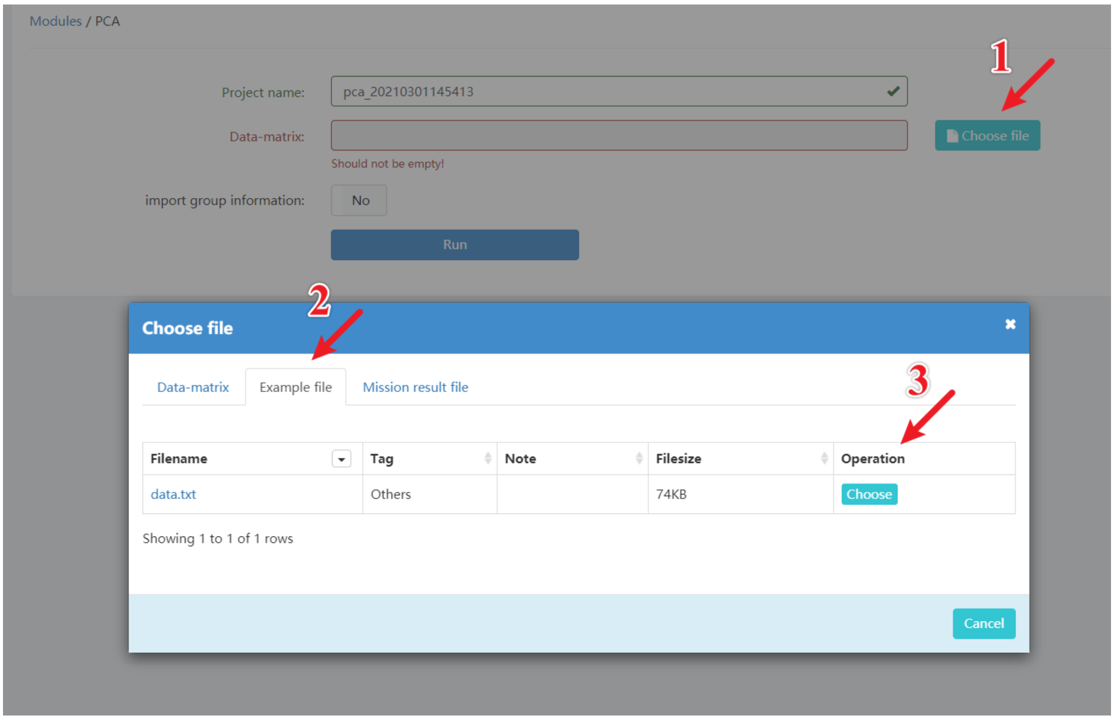

Figure S2C. Example input data in Data-repositories.

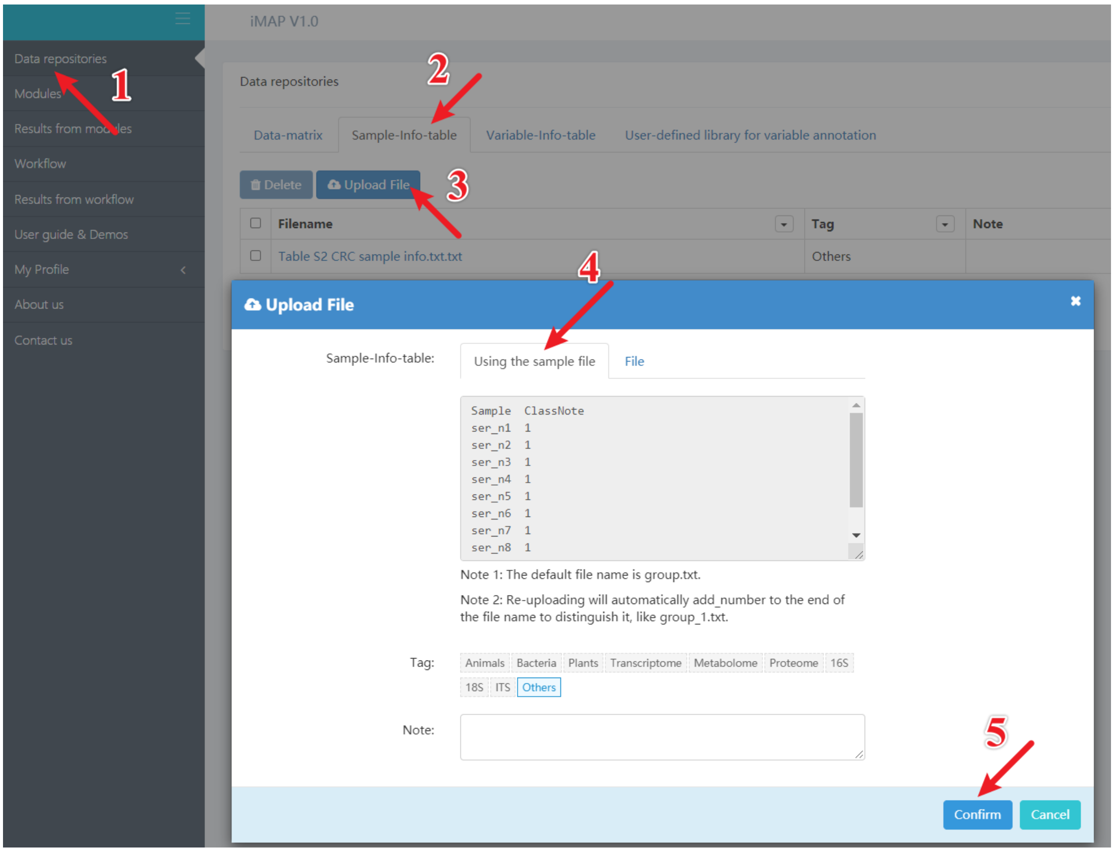

Figure S2. Three access to the example input data for every user. **(A)** Example input data for workflow construction. **(B)** Example input data for each module. **(C)** Example input data in "Data repositories" in the main menu.

Figure S3A. Workflow for predictive model building.

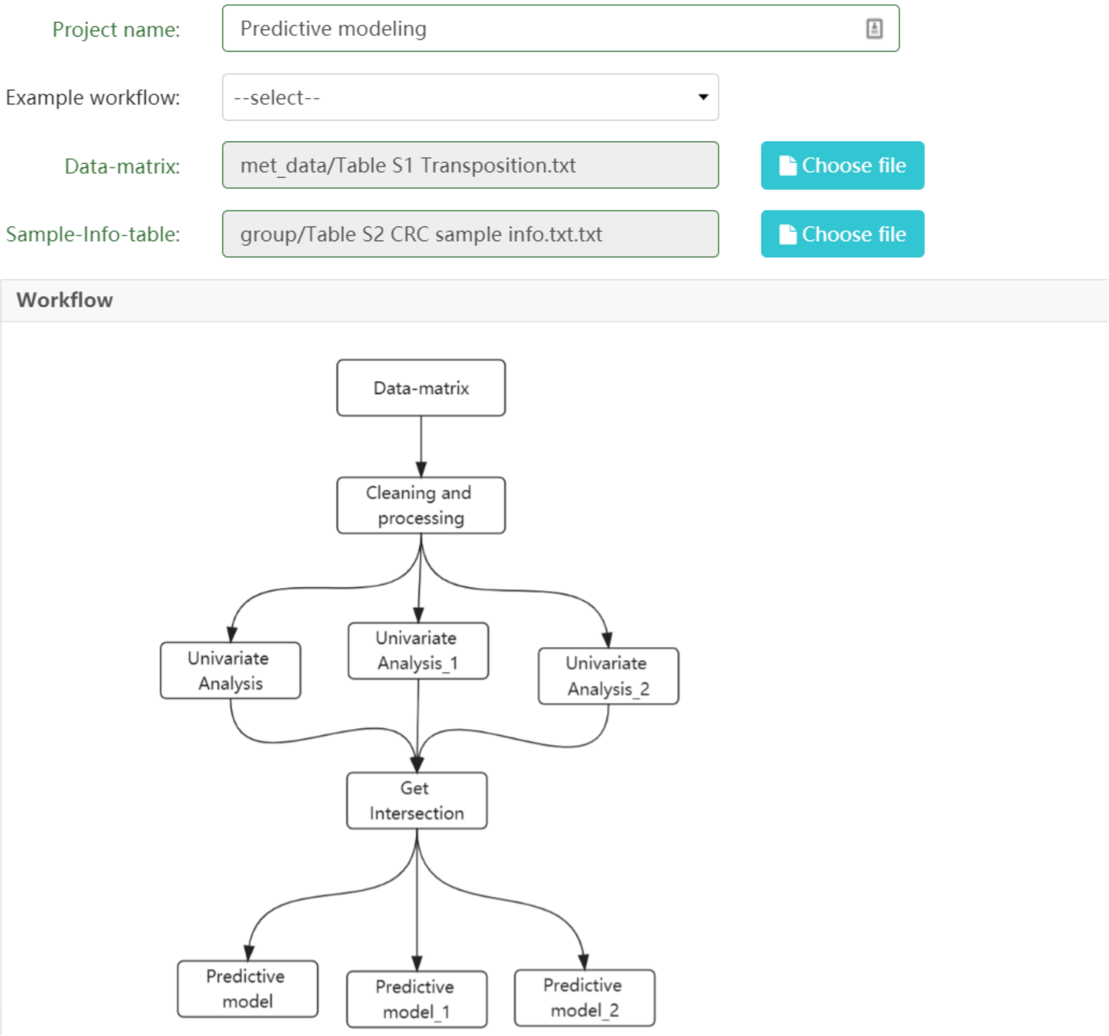

Figure S3B. Parameters utilized in the "Univariate analysis" module.

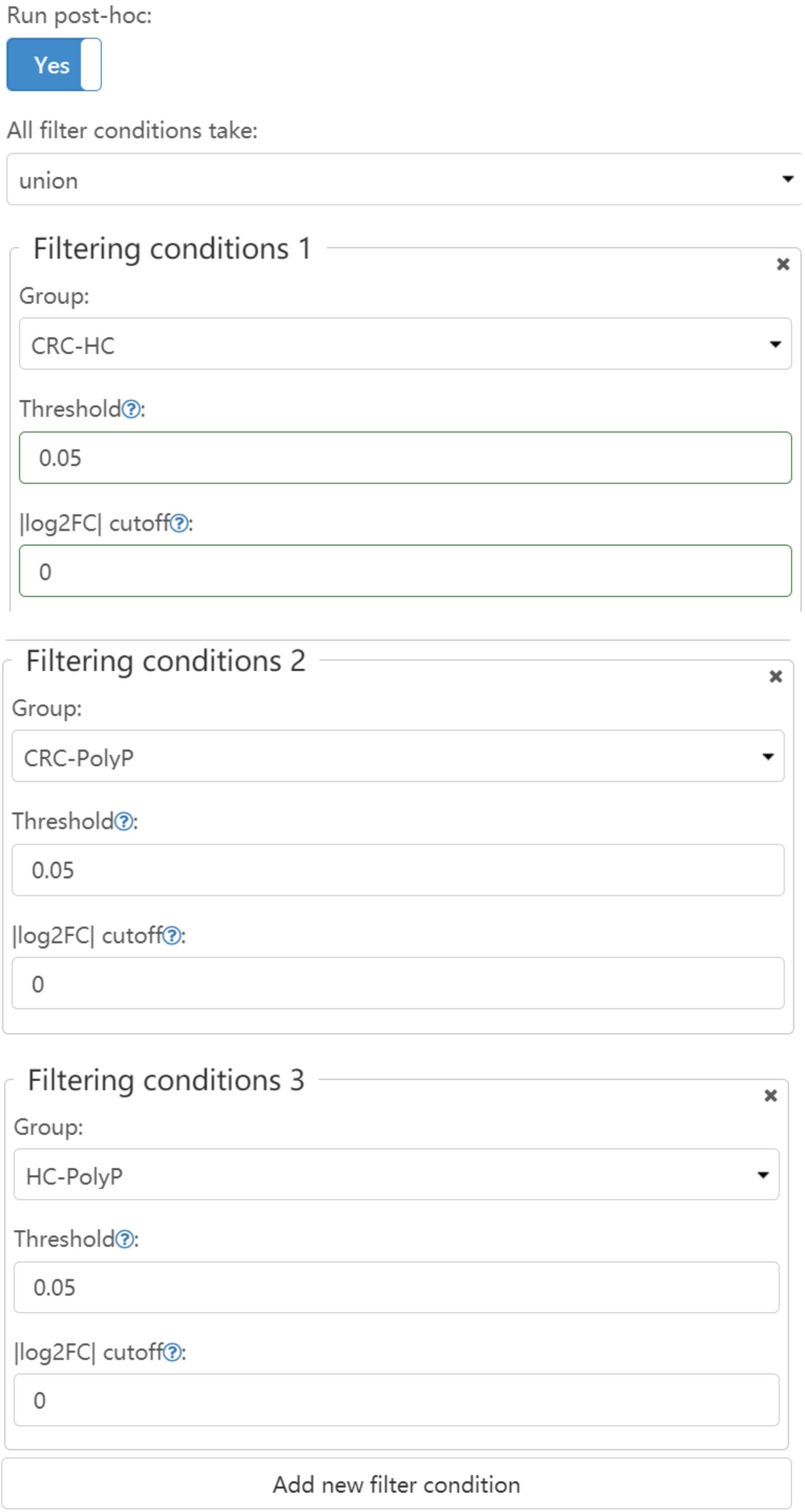

Figure S3C. Parameters utilized in the "Univariate analysis_1" module.

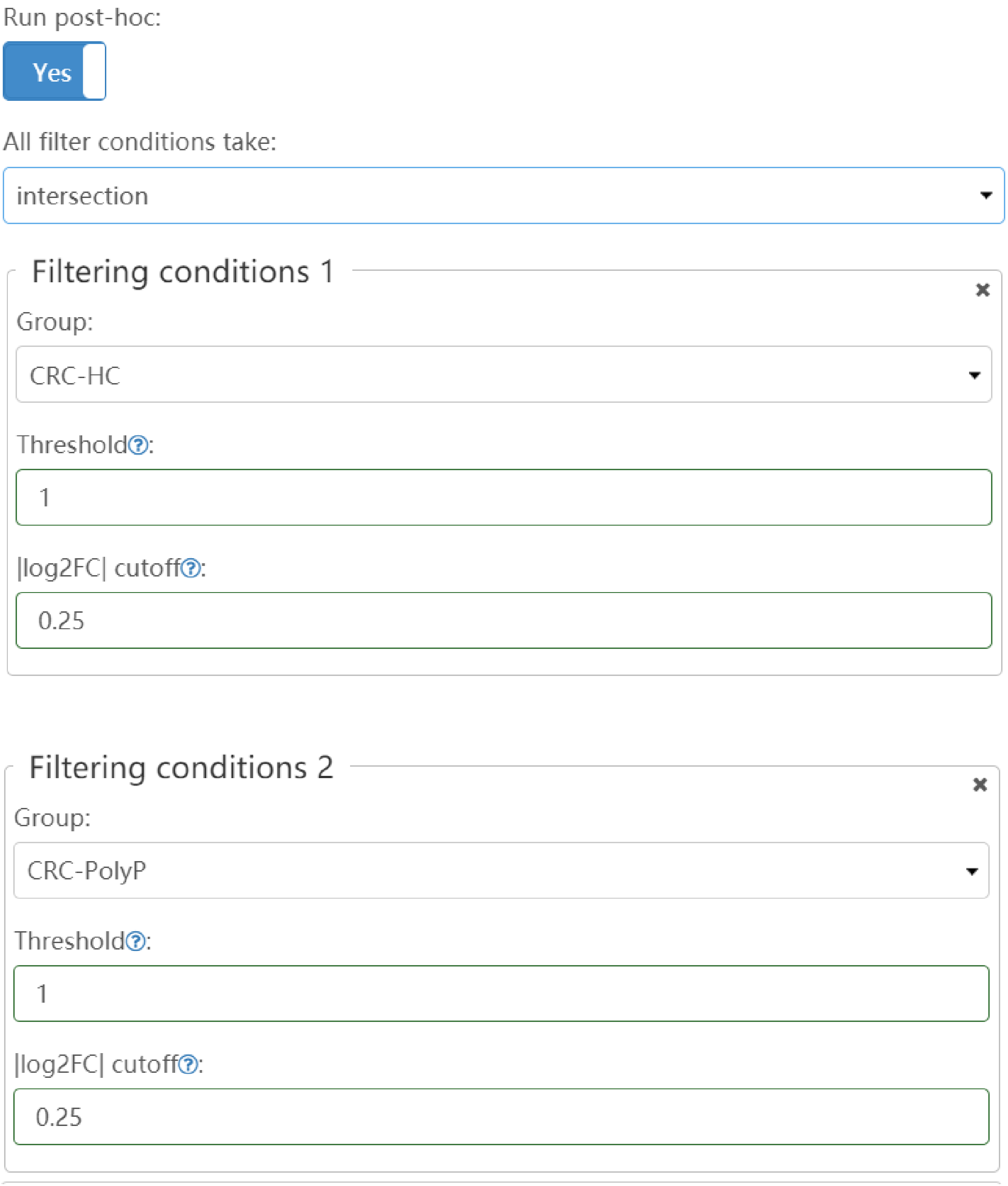

Figure S3D. Parameters utilized in the "Univariate analysis_2" module.

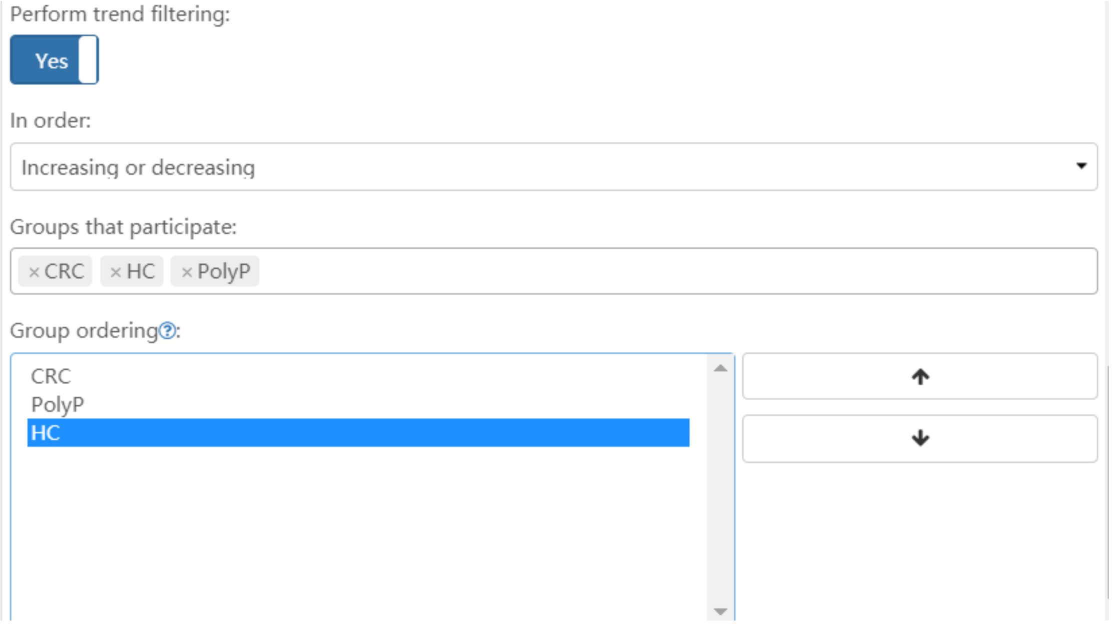

Figure S3E. Parameters utilized in the predictive modeling.

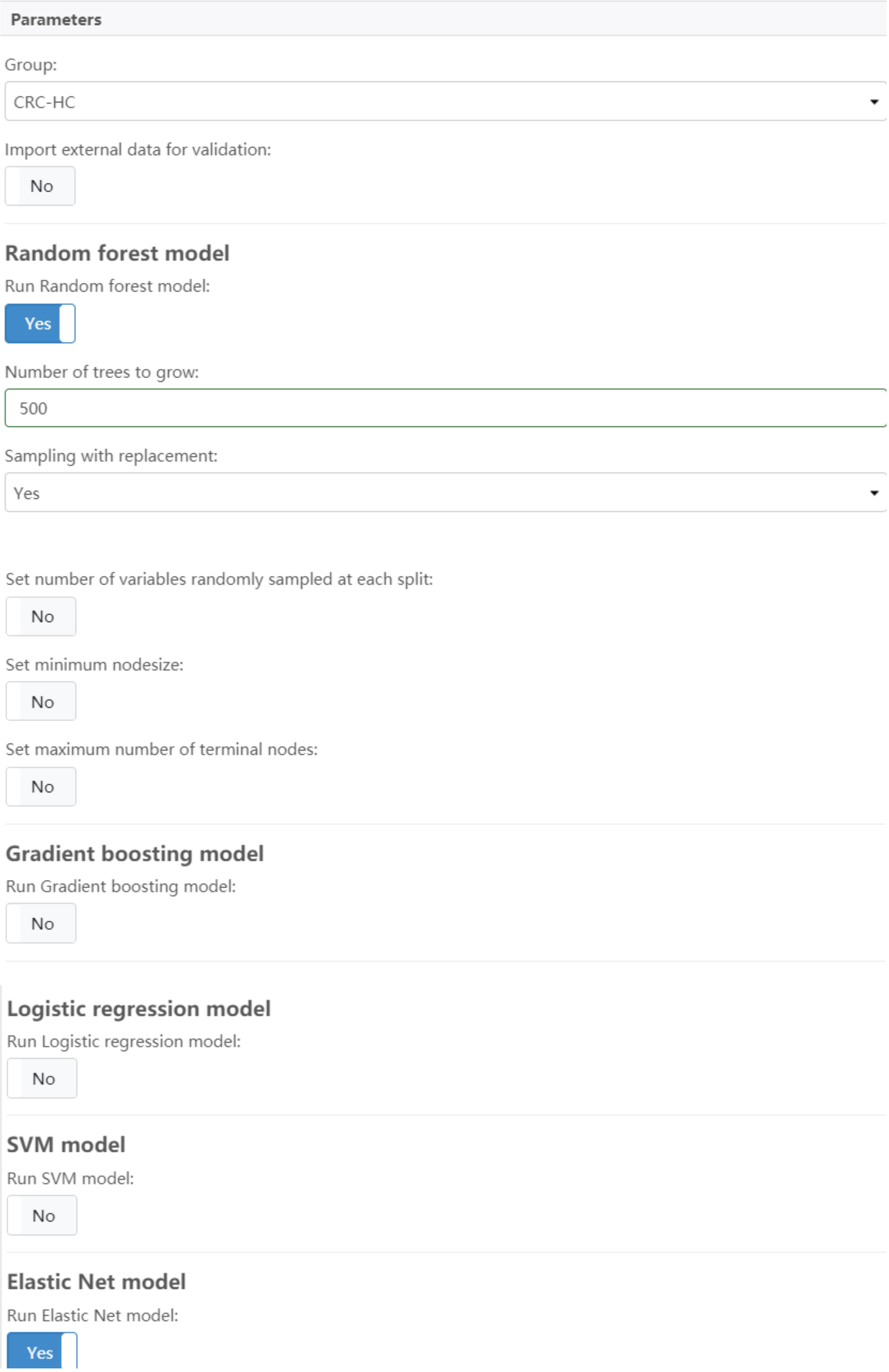

Figure S3. Workflow for predictive model building with the targeted serum metabolomic data. **(A)** Workflow overview. Three corresponding univariate analysis modules were applied to the three criteria in the metabolites selection separately. A get intersection module was used to combine the selected metabolites. **(B)** Parameters utilized in univariate analysis module to select metabolites with Pairwise post-hoc P-value < 0.05 between ≥1 group pair by Tukey test after Kruskal Wallis test. **(C)** Parameters utilized in "Univariate analysis_1" module to select metabolites with |log2FC| > 0.25 between CRC patients and Polyp patients, and |log2FC| > 0.25 between CRC patients and healthy controls. Threshold (Post-hoc P-value threshold) was set as 1 to skip post-hoc P-value selection and use |log2FC| only. **(D)** Parameters utilized in the "Univariate analysis_2" module to select metabolites ascending or descending trends in group HC, group PolyP, and group CRC. **(E)** Parameters utilized in predictive modeling. Different group pairs were chosen in the three predictive modules separately.

Figure S4A. OOB error rate plot (CRC-PolyP).

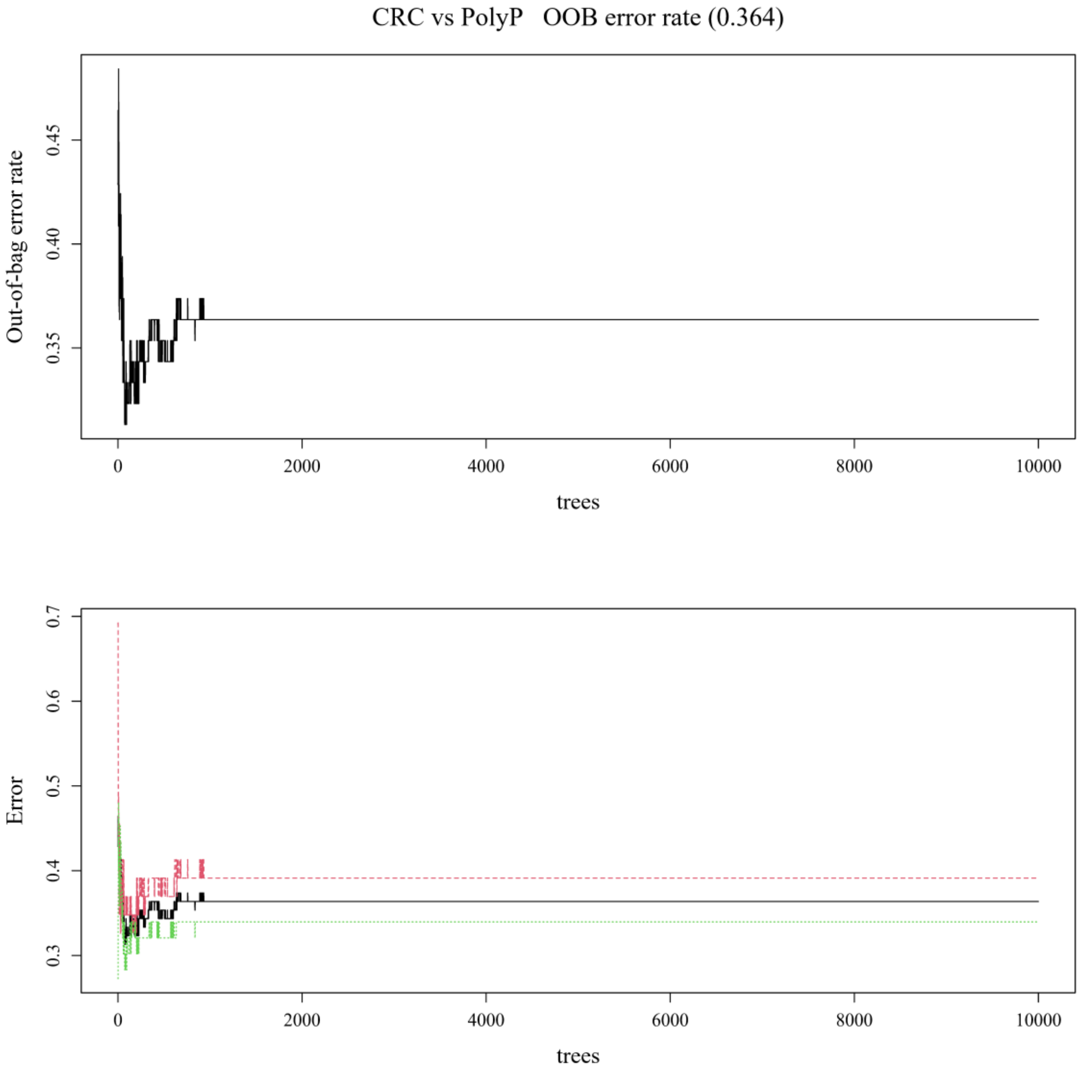

Figure S4B. OOB error rate plot (CRC-HC).

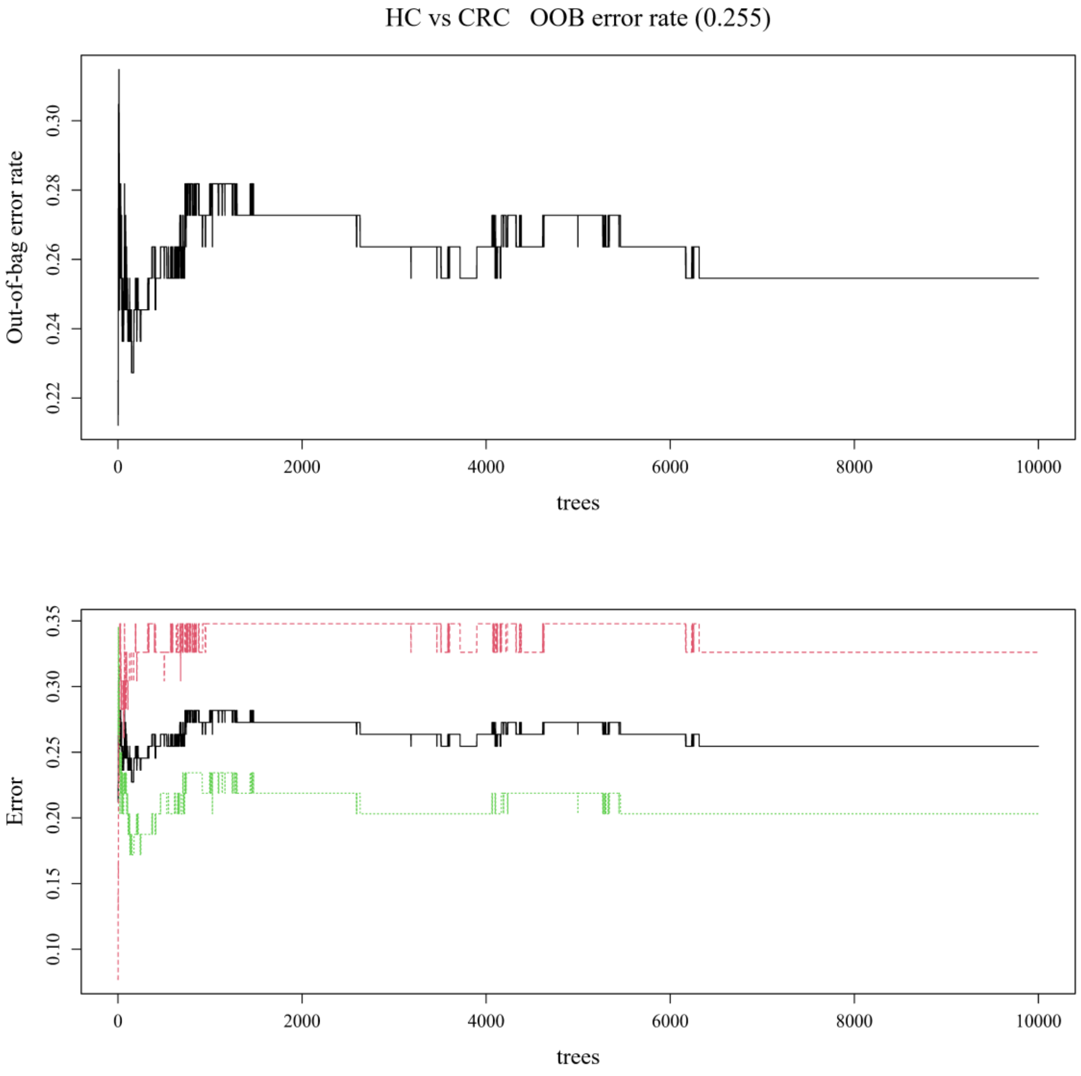

Figure S4C. OOB error rate plot (PolyP-HC).

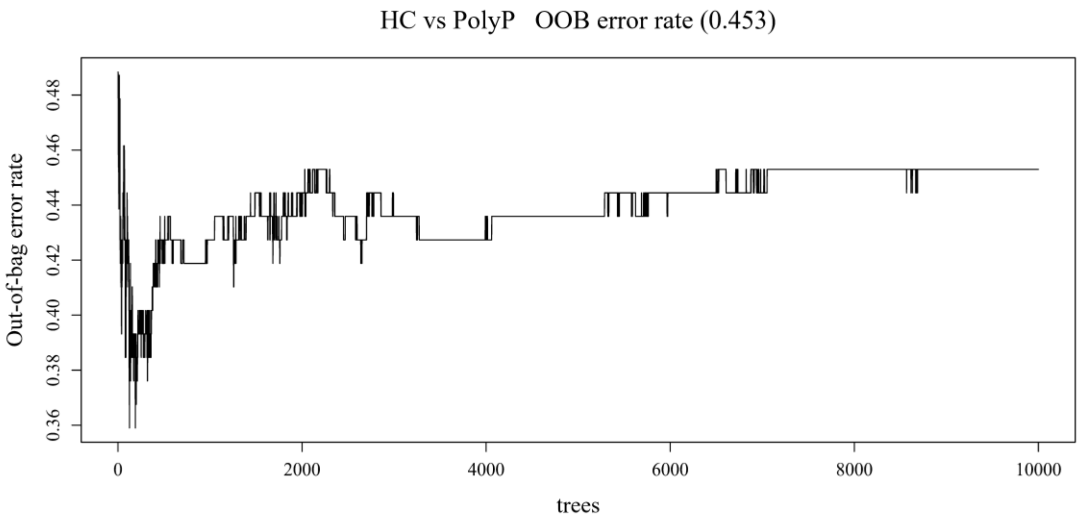

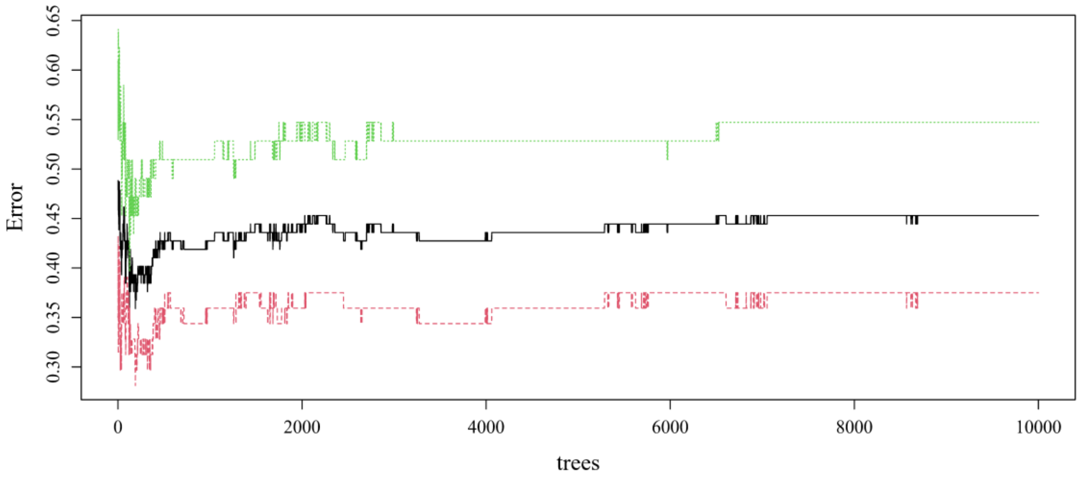

Figure S4. OOB error rates Plot between **(A)** CRC-PolyP, **(B)** CRC-HC, and **(C)** PolyP-HC, respectively. OOB error rates were calculated by R package randomForest.

Figure S5. Parameters for correlation analysis.

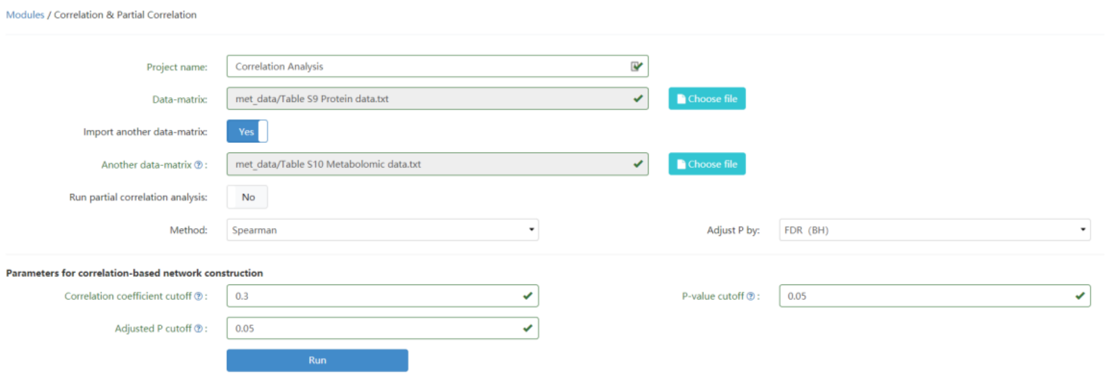

Figure S5. Parameters for correlation analysis. Spearman correlation analysis between metabolomic data and proteome data was performed by the "Correlation & Partial Correlation" module. Absolute value of correlation coefficient > 0.3, P-value cutoff < 0.05, and FDR < 0.05 (adjusted P cutoff) were used to select qualified correlation pairs.

References

Adam, M.G., Beyer, G., Christiansen, N., Kamlage, B., Pilarsky, C., Distler, M., et al. (2021). Identification and validation of a multivariable prediction model based on blood plasma and serum metabolomics for the distinction of chronic pancreatitis subjects from non-pancreas disease control subjects. *Gut*. doi: 10.1136/gutjnl-2020-320723.

Bowerman, K.L., Rehman, S.F., Vaughan, A., Lachner, N., Budden, K.F., Kim, R.Y., et al. (2020). Disease-associated gut microbiome and metabolome changes in patients with chronic obstructive pulmonary disease. *Nat Commun* 11(1)**,** 5886. doi: 10.1038/s41467-020-19701-0.

Bushman, F.D., Conrad, M., Ren, Y., Zhao, C., and Baldassano, R. (2020). Multi-omic Analysis of the Interaction between Clostridioides difficile Infection and Pediatric Inflammatory Bowel Disease. *Cell Host & Microbe*.

Chen, Z., Han, S., Zhang, J., Zheng, P., Liu, X., Zhang, Y., et al. (2021). Exploring urine biomarkers of early health effects for occupational exposure to titanium dioxide nanoparticles using metabolomics. *Nanoscale* 13(7)**,** 4122-4132. doi: 10.1039/d0nr08792k.

Liu, H., Chen, X., Hu, X., Niu, H., Tian, R., Wang, H., et al. (2019). Alterations in the gut microbiome and metabolism with coronary artery disease severity. *Microbiome* 7(1)**,** 1-14.

Liu, R., Hong, J., Xu, X., Feng, Q., Zhang, D., Gu, Y., et al. (2017). Gut microbiome and serum metabolome alterations in obesity and after weight-loss intervention. *Nature medicine* 23(7)**,** 859.

Liu, Z., Liu, M., Fan, M., Pan, S., Li, S., Chen, M., et al. (2021). Metabolomic-proteomic combination analysis reveals the targets and molecular pathways associated with hydrogen sulfide alleviating NAFLD. *Life Sciences* 264**,** 118629.

Oh, T.G., Kim, S.M., Caussy, C., Fu, T., Guo, J., Bassirian, S., et al. (2020). A universal gut-microbiome-derived signature predicts cirrhosis. *Cell metabolism* 32(5)**,** 878-888. e876.

Smilde, A.K., van der Werf, M.J., Bijlsma, S., van der Werff-van der Vat, B.J.C., and Jellema, R.H. (2005). Fusion of Mass Spectrometry-Based Metabolomics Data. *Analytical Chemistry* 77(20)**,** 6729-6736. doi: 10.1021/ac051080y.

Sreekumar, A., Poisson, L.M., Rajendiran, T.M., Khan, A.P., Cao, Q., Yu, J., et al. (2009). Metabolomic profiles delineate potential role for sarcosine in prostate cancer progression. *Nature* 457(7231)**,** 910-914. doi: 10.1038/nature07762.

Want, E.J., Wilson, I.D., Gika, H., Theodoridis, G., Plumb, R.S., Shockcor, J., et al. (2010). Global metabolic profiling procedures for urine using UPLC-MS. *Nat Protoc* 5(6)**,** 1005-1018. doi: 10.1038/nprot.2010.50.

Wozniak, J.M., Mills, R.H., Olson, J., Caldera, J.R., Sepich-Poore, G.D., Carrillo-Terrazas, M., et al. (2020). Mortality Risk Profiling of Staphylococcus aureus Bacteremia by Multi-omic Serum Analysis Reveals Early Predictive and Pathogenic Signatures. *Cell* 182(5)**,** 1311-1327.e1314. doi: 10.1016/j.cell.2020.07.040.

Xuan, Q., Ouyang, Y., Wang, Y., Wu, L., Li, H., Luo, Y., et al. (2020). Multiplatform Metabolomics Reveals Novel Serum Metabolite Biomarkers in Diabetic Retinopathy Subjects. *Adv Sci (Weinh)* 7(22)**,** 2001714. doi: 10.1002/advs.202001714.
